# Recurrent, Robust and Scalable Patterns Underlie Human Approach and Avoidance

**DOI:** 10.1371/journal.pone.0010613

**Published:** 2010-05-26

**Authors:** Byoung Woo Kim, David N. Kennedy, Joseph Lehár, Myung Joo Lee, Anne J. Blood, Sang Lee, Roy H. Perlis, Jordan W. Smoller, Robert Morris, Maurizio Fava, Hans C. Breiter, for the Phenotype Genotype Project in Addiction and Mood Disorders (PGP)

**Affiliations:** 1 Motivation and Emotion Neuroscience Collaboration (MENC), Athinoula A. Martinos Center for Biomedical Imaging, Department of Radiology, Massachusetts General Hospital and Harvard Medical School, Boston, Massachusetts, United States of America; 2 Laboratory of Neuroimaging and Genetics, Department of Psychiatry, Massachusetts General Hospital and Harvard Medical School, Boston, Massachusetts, United States of America; 3 Center for Morphometric Analysis, Department of Neurology, Massachusetts General Hospital and Harvard Medical School, Boston, Massachusetts, United States of America; 4 Department of Bioinformatics, Boston University, Boston, Massachusetts, United States of America; 5 Mood and Motor Control Laboratory, Department of Psychiatry, Massachusetts General Hospital and Harvard Medical School, Boston, Massachusetts, United States of America; 6 Depression Clinic and Research Program, Department of Psychiatry, Massachusetts General Hospital and Harvard Medical School, Boston, Massachusetts, United States of America; 7 Psychiatric and Neurodevelopmental Genetics Unit of the Center for Human Genetic Research, Massachusetts General Hospital and Harvard Medical School, Boston, Massachusetts, United States of America; Kyushu University, Japan

## Abstract

**Background:**

Approach and avoidance behavior provide a means for assessing the rewarding or aversive value of stimuli, and can be quantified by a keypress procedure whereby subjects work to increase (approach), decrease (avoid), or do nothing about time of exposure to a rewarding/aversive stimulus. To investigate whether approach/avoidance behavior might be governed by quantitative principles that meet engineering criteria for lawfulness and that encode known features of reward/aversion function, we evaluated whether keypress responses toward pictures with potential motivational value produced any regular patterns, such as a trade-off between approach and avoidance, or recurrent lawful patterns as observed with prospect theory.

**Methodology/Principal Findings:**

Three sets of experiments employed this task with beautiful face images, a standardized set of affective photographs, and pictures of food during controlled states of hunger and satiety. An iterative modeling approach to data identified multiple law-like patterns, based on variables grounded in the individual. These patterns were consistent across stimulus types, robust to noise, describable by a simple power law, and scalable between individuals and groups. Patterns included: (i) a preference trade-off counterbalancing approach and avoidance, (ii) a value function linking preference intensity to uncertainty about preference, and (iii) a saturation function linking preference intensity to its standard deviation, thereby setting limits to both.

**Conclusions/Significance:**

These law-like patterns were compatible with critical features of prospect theory, the matching law, and alliesthesia. Furthermore, they appeared consistent with both mean-variance and expected utility approaches to the assessment of risk. Ordering of responses across categories of stimuli demonstrated three properties thought to be relevant for preference-based choice, suggesting these patterns might be grouped together as a relative preference theory. Since variables in these patterns have been associated with reward circuitry structure and function, they may provide a method for quantitative phenotyping of normative and pathological function (e.g., psychiatric illness).

## Introduction

Intentional behavior, across a spectrum of healthy and disordered conditions such as addiction, is hypothesized to reflect differences in judgment and decision-making around relative preference [1]. Relative preference is defined by the variable extent an individual will approach or avoid [2]–[4] commodities and events based on their rewarding or aversive features [5], [6]. It can be expressed by the payment an individual makes to avoid a perceived bad outcome, or approach a positive one. Consumers undertake such transactions to optimize their utility (i.e., overall satisfaction or well-being) based on relative preferences [7]–[11]. Prior study of relative preference (with variable degrees of uncertainty) has calibrated ratings of personal utility against a global framework such as the macroeconomic pricing of commodities. This calibration has produced a value function that is recurrent and grounds modern prospect theory [12], [13]. Prospect theory informs us that subjective value or relative preference is modeled by a value function that is convex for losses, concave for gains, and steeper for losses than gains. This value function is modulated by probabilistic information [7], [12], [13].

**Figure 1:**
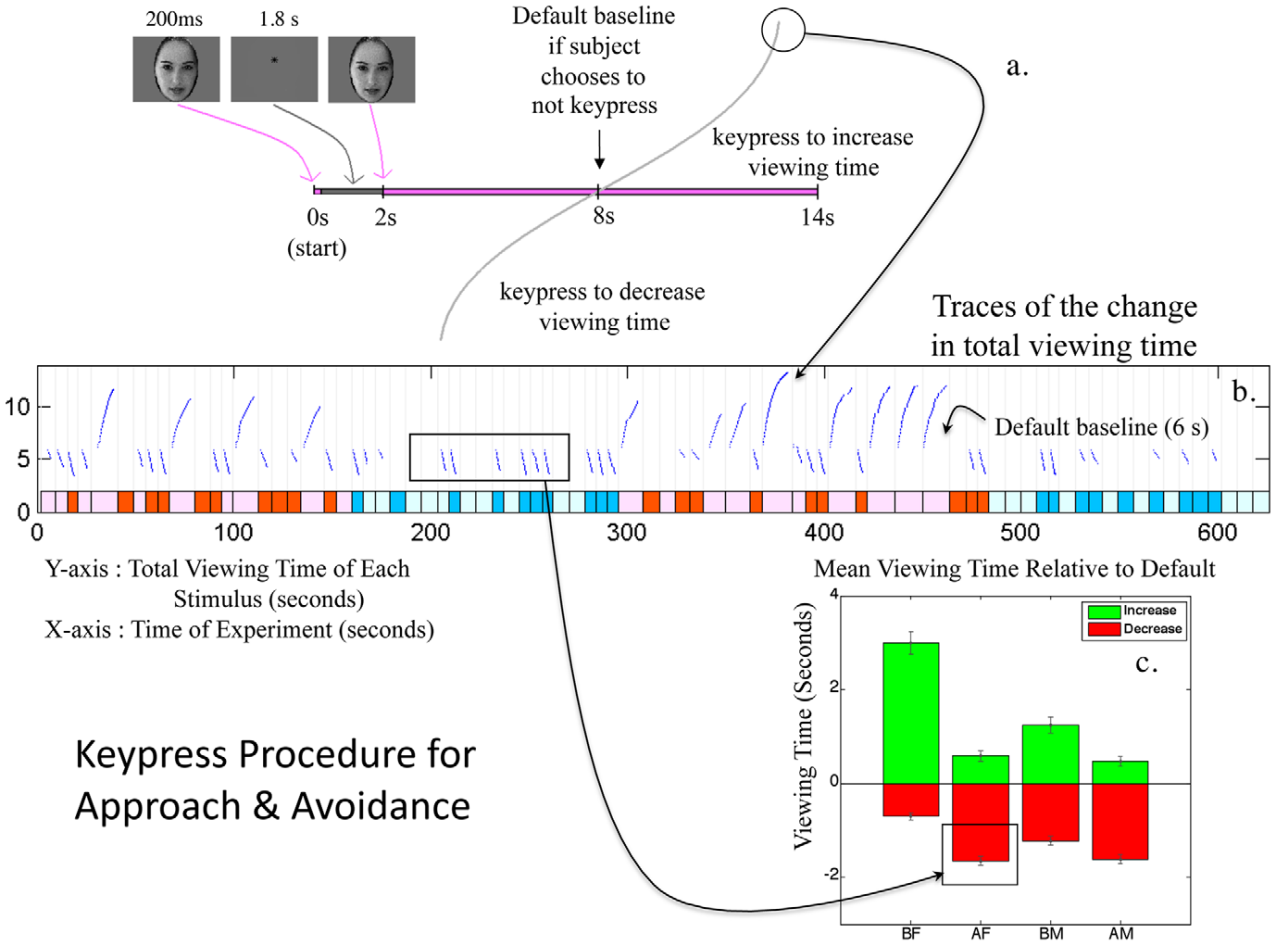
Experimental Design of Keypress Procedure. (**a**) This schematic illustrates the four potential responses to the stimuli: to increase, decrease, variably increase or decrease for the same image, or accept the default viewing time of 6 (+2) seconds. The default condition controls for subjects having an intention to keypress and alter viewing time, but not acting on this intention. (**b**) The traces of individual keypressing behavior to each picture are shown for an anonymous subject. Time intervals are color coded by experimental condition as follows: beautiful female (pink), average female (red), beautiful male (light blue), and average male (dark blue). Stimuli presentation was segregated by gender so that beautiful female faces did not bias all other responses (i.e., responses to male faces). Each blue trace of actual keypress data is shown relative to the default baseline for viewing. (**c**) Viewing time relative to the default time (location and standard errors) for the ensemble of BF, AF, BM, AM faces are shown as a bar graph.

Approach and avoidance behavior can also be modeled by data from a validated keypress procedure [14]–[21] that is used within an intrinsic motivation-like framework in which no external rewards are provided [22], [23], yet participants can produce variable amounts of work [24], [25] to modulate the time of stimulus viewing. As a variant of approaches used to study effort-based decision-making [26]–[28] the keypress procedure appears to quantify (i) decision-making regarding the valence of behavior (i.e., positive valence = approach, and negative valence = avoidance) and (ii) judgments determining its magnitude (Figure 1) [15], [20], [29]. These analogies aside, this procedure is not easily connected to a global framework pricing commodities and other behavioral economic constructs as it operates only within a person- or agent-centric context. Keypress measures of approach and avoidance can be connected to neural systems [14]–[16], [18]–[20], as has been done with prospect theory [30], [31], and represent an important methodology for bridging animal and human research of reward/aversion processing [14], [32] and neuroeconomics [10], [11], [33]–[37].

Despite research to date, we do not know if there is a balance or “trade-off” between keypress measures of approach and avoidance. We do not know if there are “limits” to approach and avoidance keypressing analogous to the saturation observed in neurophysiology with variance-mean relationships whereby the graph ramps up to a maximum and then returns to baseline minimum [38]. We also do not know how approach and avoidance might interact to produce lawful patterns underlying valuation as observed with prospect theory [7], [12] or other theories such as the matching law [39]–[41]. Are there patterns to approach and avoidance that meet engineering criteria for lawfulness and are consistent with established features of reward/aversion behavior such as (i) the overweighting of losses relative to gains [7], [13], [31], (ii) the relative apportionment or allocation of behavior between two categories of reinforcement [39], [40], [42], or (iii) alliesthesia or hedonic deficit effects [43]–[46]? These questions framed *the goal* for this work of determining whether approach/avoidance behavior is governed by quantitative principles that meet engineering criteria for lawfulness, and encoded by known features of reward/aversion function. Such findings would have relevance for quantitative phenotyping and subtyping of psychiatric disorders [47]–[49] that have been connected to reward/aversion circuitry [5], [8], [20], [31], [33], [35]–[37], [50]–[52] such as addiction [53]–[56] and major depressive disorder [57]–[59].

To meet this goal, we performed three sets of experiments in three distinct cohorts of healthy subjects, and analyzed the data following an iterative modeling schema adapted from Banks and Tran [60]. These model-free procedures were first applied to data collected while viewing pictures of “beautiful” and “average” faces (Supporting Information Figure 1 or Figure S1) [14]. Behavioral patterns observed with beauty stimuli were then tested for recurrency using two other stimulus sets, including (a) a well-validated stimulus set, the International Affective Picture System (IAPS) [61], [62] (Figure S2) in an independent cohort of subjects, and (b) a stimulus set of food pictures tested during conditions of hunger and satiation in a third cohort of healthy subjects (Figure S3). The iterative modeling approach sought to (a) identify quantitative patterns between variables describing approach and avoidance, (b) determine if these patterns met criteria for recurrency (i.e., consistency across stimuli), robustness to noise, and scalability, (c) characterize whether these patterns were consistent with established features of reward/aversion function, and (d) rule out any experimental confounds to these patterns, including the possibility of trial-by-trial interactions [63].

These procedures resulted in observation and validation of a set of patterns underlying human approach and avoidance that are law-like, and are consistent with critical features of prospect theory, the matching law, and alliesthesia. These patterns appear to scale between groups and individuals.

## Results

The keypress task was first conducted using a stimulus set associated with strong reward/aversion behavior, namely a picture set of faces of men and women who were models or non-models [i.e., beautiful female (BF), average female (AF), beautiful male (BM), average male (AM) faces [14]. Any relationships found to be recurrent for all four conditions in the “beauty” stimulus set were subsequently tested for recurrency using two other stimulus sets in separate cohorts of subjects. Starting with data from the beauty keypress experiments, we graphed the relationship between approach and avoidance measures (using a number of variables described in the next paragraph) to determine if a “trade-off” or “limit” function could be identified. We then tested relationships between (i) trade-off plot variables and (ii) independent variables related to keypress intensity, which might share features with established theories of valuation. We assessed the mathematical fit of any graphical structure (i.e., pattern) observed, how this fit scaled between group and individual data, and whether the structure demonstrated the signature of a power law. Graphical structure was compared to simulated hypothetical data to rule out that any observed structure was mathematically trivial, and any trade-off plots were tested for robustness against noise. A number of control analyses were also performed to facilitate interpretation of findings. One such control analysis sought to assure that keypress responses for any one picture were not influencing subsequent keypress responses to other pictures through an analysis of variance of trial-by-trial interactions.

For these analyses, we assessed a range of descriptive statistical variables. Location measures included mean, median, and mode of positive (approach) and negative (avoidance) keypressing, along with maximum and minimum values in the data set. Dispersion estimates included standard deviation and median-absolute deviation. Given these dispersion estimates take into account the range of responses around a reference point, and not the pattern of response within each experimental variable, such measures may not be sensitive to qualitatively different behavioral patterns. Entropy, signal-to-noise, and covariance variables were, thus, assessed since they quantify the characteristics of response pattern (e.g. the extent of irregularity/heterogeneity [64]–[66]) produced by underlying behavioral microvariables, and would therefore be more sensitive to patterns in approach and avoidance.

### Approach/Avoidance Trade-offs

For group data, no consistent pattern was observed in the graphs between location measures (e.g., mean, median, mode, minimum, maximum) of positive (approach) and negative (avoidance) keypressing across the four categories of faces (i.e., BF, AF, BM, AM faces). Nor were graphical patterns (e.g., manifold, function or envelope) observed for group data with the standard deviation and median-absolute deviation. Absent patterns at the level of group data for these variables, linear fitting of individual data suggested patterns across the four experimental conditions (i.e., categories of faces) for both the mean 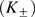 and standard deviation variables 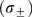. These patterns, though, displayed significant heterogeneity (i.e., inconsistency), with a broad range of values and both positive and negative slopes for subsets of individuals. Hence, 12 of 77 subjects had positive slopes for 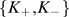 graphs, with a mean of 1.16±2.11 and range of 5.50 (or an angle of 79.7° between minimum and maximum), whereas 65 of 77 subjects had negative slopes for 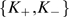 graphs, with a mean of −2.85±1.98 and range of 11.21 (or an angle of 81.5° between minimum and maximum). For 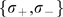 graphs, 50 of 77 had positive slopes, with a mean of 3.41±3.04 and range of 14.56 (or an angle of 86.1° between minimum and maximum), whereas 27 of 77 had negative slopes, with a mean of −3.48±3.42 and range of 17.10 (or an angle of 82.7° between minimum and maximum).

**Figure 2:**
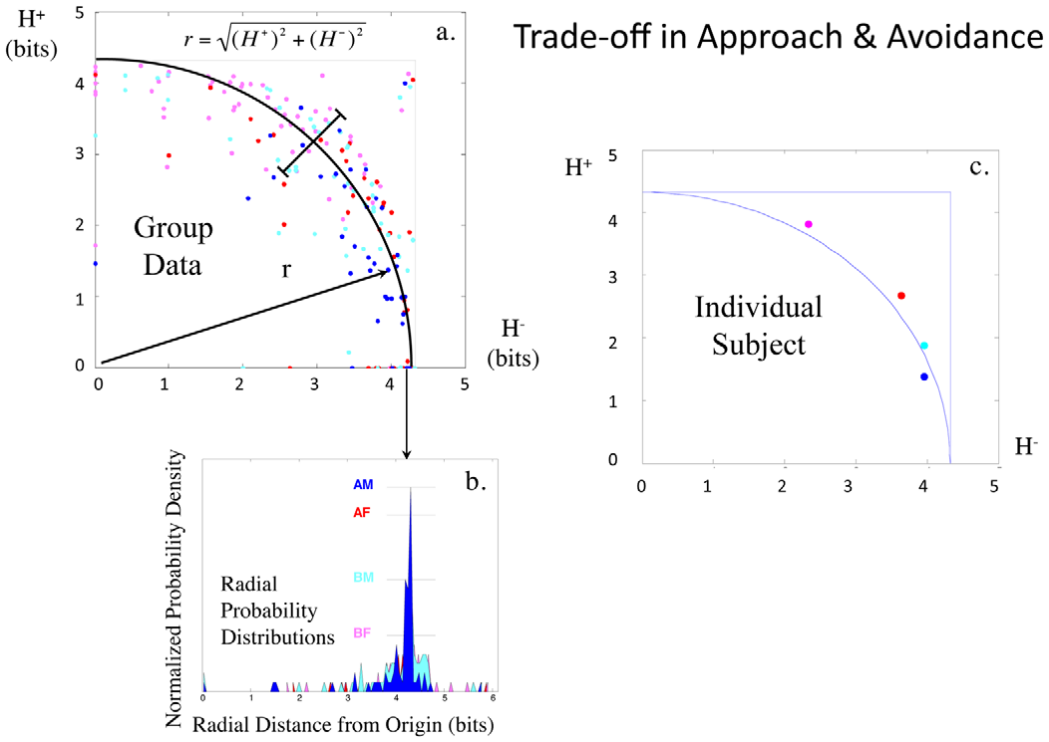
Preference Uncertainty Trade-off. (**a**) shows a graph of 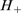 (y axis) vs. 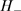 (x axis) for BF, AF, BM, AM faces in 77 healthy control subjects [experimental conditions (i.e., stimulus categories) are color coded as indicated in (b)]. The central tendency of the 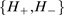 manifold is approximated by a black quarter-circle, with its dispersion via crossbars and mathematical formulation as 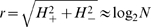, where N = the number of items in the experimental condition. Spectra for the radial probability distributions of responses to the BF, AF, BM, AM faces are superimposed in (**b**). Given 20 items for each set of faces, this plot produces a distribution centered on 4.32 bits. In (**c**), the 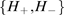 data for four categories of faces are plotted for one individual.

In contrast to these results with location and dispersion variables, consistent patterns were observed for graphs with group data using pattern variables such as (i) signal to noise ratios 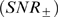, (ii) covariance estimates 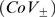, and (iii) Shannon entropy 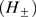 estimates [67] (Figure 2a; Figure S4). All of these patterns for 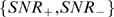, 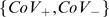, and 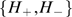 plots were recurrent across BF, AF, BM, and AM face stimuli (representing one simplex manifolds for 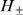 and 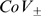 estimates, and a boundary envelope for the 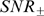 estimate). Spectra for the radial distribution of the 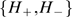 graphs for the BF, AF, BM, and AM faces exhibited similar central tendencies when superimposed (Figure 2b), and were amenable to Gaussian fitting, although t location-scale fitting was the most accurate (Figure S5; Supporting Information File S1 Section I). These 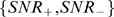, 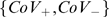, and 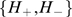 patterns were present both with keypress data and with total view time data (Figure S6), ruling out resistive function effects.

**Figure 3 pone-0010613-g003:**
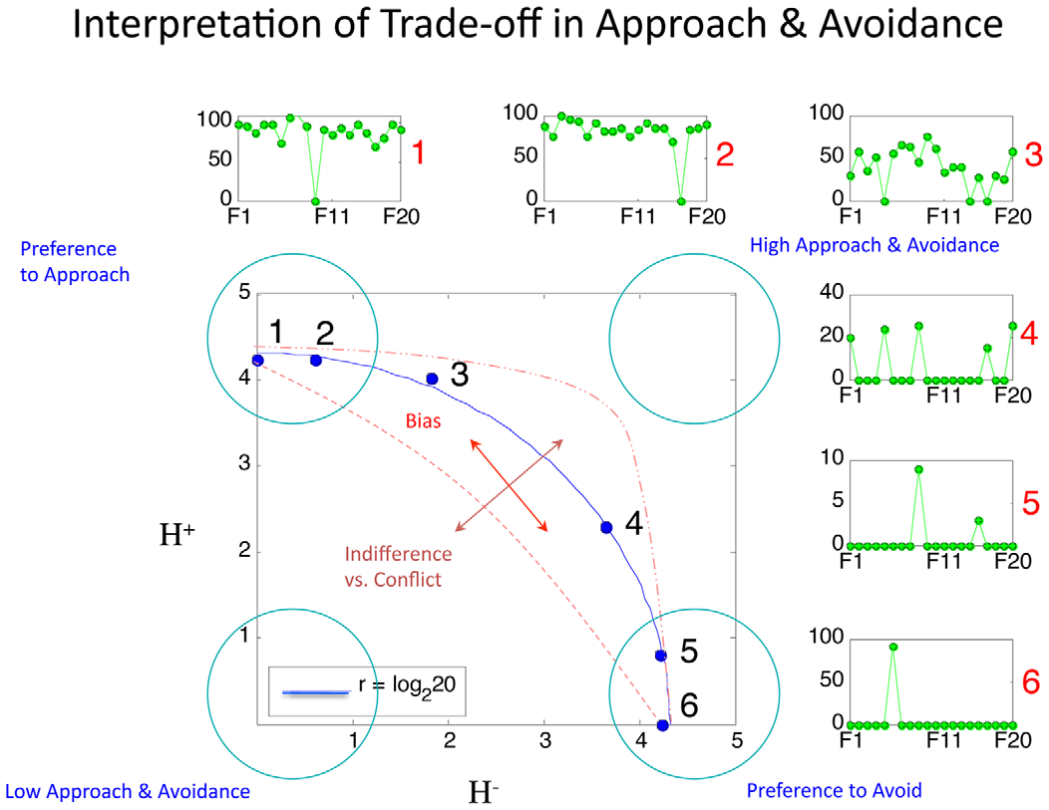
Interpretation of Trade-off Plot. This cartoon provides an example of possible keypress patterns that fall at six different positions on the 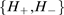 manifold, using data from six subjects toward the same 20 BM faces (F1–F20) for increasing viewing time (data are shown for approach only). For the six approach graphs shown, the x-axis represents the 20 faces in an experimental condition (i.e. BF, AF, BM, AM), and the y-axis represents the number of keypresses toward that face picture. The Shannon entropy was computed using data in this format (see Methods, Analyses, *Descriptive Statistical Measures*). To schematize the balance of approach 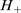 and avoidance 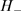, one might imagine a matching of graphs #1 with #6, #2 with #5, and #3 with #4, where one graph represents the keypress responses for approach 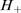 and the other avoidance 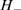. For the purposes of illustration, we assigned zero values here to 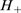 in sub-figure #6. For each sub-figure (#1–6) above and to the right of the 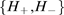 manifold, data has been auto-scaled to optimize the pattern display. Overall, this graph represents relative approach or avoidance bias along the polar angle, whereas the extent of indifference/conflict an individual feels toward an experimental condition (i.e. BF, AF, BM, or AM) is distributed in radial fashion from the origin.

The mathematical description of the 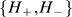 plot was the simplest of the three pattern variables (i.e., easiest to parameterize), with a central tendency approximated by 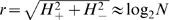, where r = radius from the origin and N = the number of pictures in the experimental condition (Figure 2a; Supporting Information File S1 Section II, Pattern 1). The mathematical descriptions of 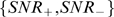, 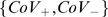, and 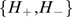 graphs were similar at the scale of the group and at the scale of the individual for the BF, AF, BM, and AM faces (Figure 2c), albeit with differing parameter fits. With either group or individual data, angular distribution along these manifolds signaled a trade-off in approach and avoidance bias (Figure 3).

Simulation of behavior limited to only approach or avoidance, and thus yoked between these two (i.e., a theoretical case in which subjects could not accept the default position or switch between approach and avoidance behaviors), showed that this response profile would approximate the inner distribution of the 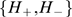 trade-off plot (Figure 3: red dashed line without dots; see Supporting Information File S1 Section III). The outer distribution of the 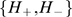 trade-off plot was approximated by variance-matched Gaussian noise (see below), and by subjects (in the right upper corner of the plot) who used both increasing and decreasing keypresses to variable extents for the same stimulus item. Individuals falling far internal to the manifold (i.e., on the H+ and H− axes, or clustered far inside the arc of r) represented individuals who accepted the default viewing time at least once.

### Other Relationships with Trade-off Variables

#### Mean Keypress Intensity (K) and Trade-off Variables

**Figure 4 pone-0010613-g004:**
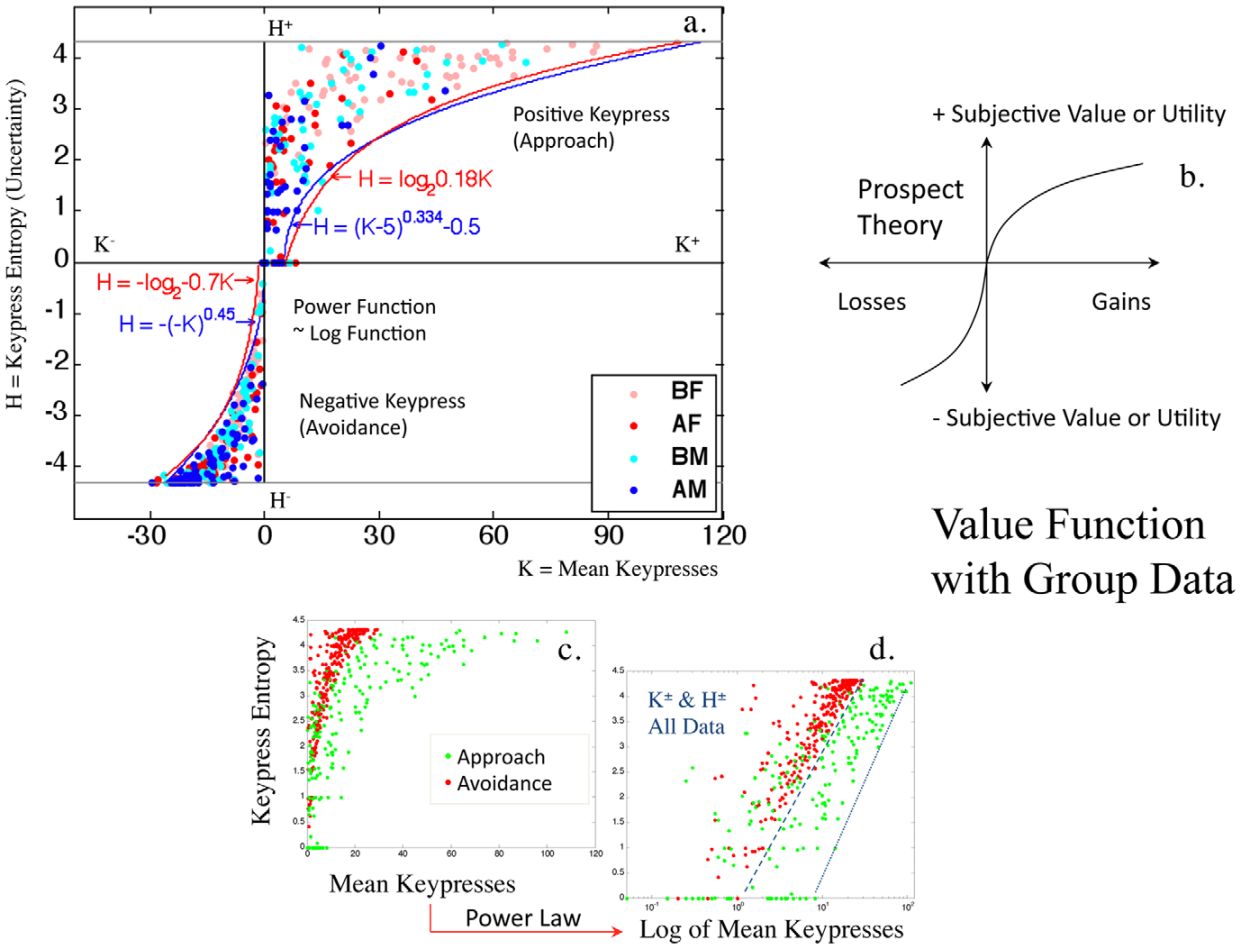
Value Function with Group Data. In (**a**), the 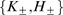 boundary envelope is shown for BF, AF, BM, AM faces in 77 healthy control subjects. The envelope can be fit well either via a logarithmic function or a power function, over the range of keypress responses. As a power function, this envelope has a similar structure to the value function in prospect theory (**b**). When approach behavior (green points) and avoidance behavior (red points) are plotted together (**c**), one can readily observe the steeper trajectory of the envelope for avoidance responses, which in prospect theory is interpreted as “loss aversion”. With transformation of the axes (**d**), both the 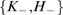 envelope and 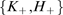 envelope show power law scaling.

Graphs of group data for 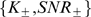, 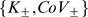, and 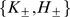 produced distributions with well-delineated envelopes (Figure 4a; Figure S7a–d), that were recurrent for BF, AF, BM, AM faces. Please see Methods, and Supporting Information File S1 Section IV (for example with 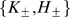) regarding the fitting of envelopes versus functions. The 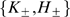 envelope resembled the value function for prospect theory (Figure 4b), in that it encoded an increased steepness for avoidance relative to approach responses, which is interpreted as “loss aversion” in prospect theory [7], [13], [31]. When the coordinate system of the 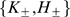 “value function” was converted to a semi-log scale (given the Shannon entropy already included a logarithmic computation), it became linear (Figure 4c,d), with the signature of a power law [68]–[70]. Linear fits of the log-transformed group data revealed an 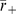 that ranged between 0.64 and 0.81, and an 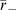 that ranged between 0.81 and 0.92 for approach and avoidance responses, respectively (Table 1). Although the 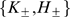 value function could be represented as a logarithmic function (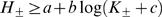) or a power function (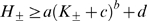) (Figure 4a; Supporting Information File S1 Section II, Pattern 2; Supporting Information File S1 Section IV), scaling the argument K by a constant factor in 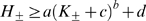 caused a proportionate scaling of H [71], [72]. Furthermore, with power law scaling, the ratios of slopes and intercept offsets for 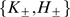 envelopes had narrow ranges across conditions (Supporting Information Table 1 or Table S1, Supporting Information File S1 Section V).

**Table 1:** Group Data for Value Function, Beauty Stimuli.

Stimulus Category	Variables	Parameter	Value
Beautiful Female	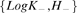	Pearson Correlation	.911
		Sig. (2-tailed)	1.127E-028
		N	72
	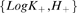	Pearson Correlation	.762
		Sig. (2-tailed)	7.115E-014
		N	67
Average Female	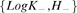	Pearson Correlation	.894
		Sig. (2-tailed)	1.879E-027
		N	76
	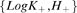	Pearson Correlation	.684
		Sig. (2-tailed)	4.471E-008
		N	50
Beautiful Male	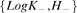	Pearson Correlation	.922
		Sig. (2-tailed)	5.862E-031
		N	73
	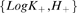	Pearson Correlation	.806
		Sig. (2-tailed)	1.425E-014
		N	59
Average Male	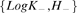	Pearson Correlation	.811
		Sig. (2-tailed)	1.83E-018
		N	74
	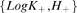	Pearson Correlation	.644
		Sig. (2-tailed)	5.562E-006
		N	41

Linear fits for the group data from 77 subjects keypressing for beautiful and average faces. Eight correlations were performed between log-transformed mean intensity data (K) and the Shannon entropy of the keypress responses (H), stratified by the stimulus condition (BF, AF, BM, AM faces) and valence of keypress (approach, +, or avoidance −). The number of subjects producing keypress data for a stimulus condition by valance of response is listed for N. Note that e-xxx denotes 10^−xxx^ for the p value.

**Figure 5 pone-0010613-g005:**
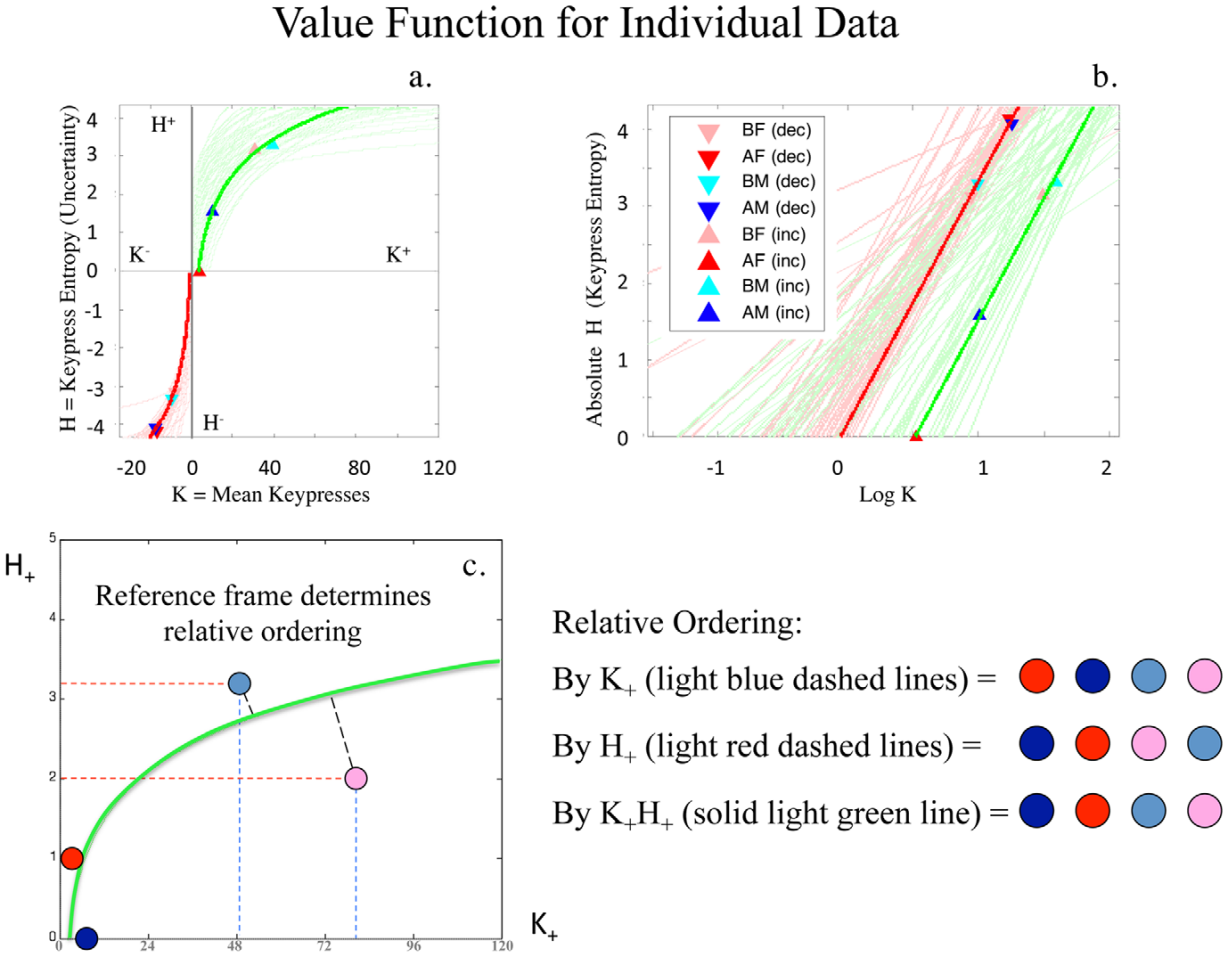
Value Function with Individual Data. In (**a**), data for the BF, AF, BM, AM faces in one individual is shown for 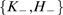 and 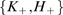 plots, superimposed on the fits for the other individuals in the cohort. With the same log transformation of axes performed for group data, one observes in each individual the signature of a power law. Here, the data for one individual is highlighted (**b**) above the graphs in lighter colors for the rest of the subjects. It is important to note that the structure of these individual plots is consistent with the respective boundary envelopes for group data. Interpreting rank order of experimental conditions on these 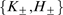 graphs depends on how one frames the measurement of relative position, (**c**). If one frames the ordering of experimental conditions by either axis (dotted blue lines for x-axis, dotted red lines for y-axis), one observes different relative orderings. A third ordering is possible if one frames the positioning relative to the power function fit for 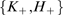 (light green line), which calibrates the pattern of responses across items in an experimental condition (H) to the mean intensity of responses (K).

The mathematical structure of the 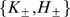 envelope for group data (defining a boundary for mappings of 77 subjects at one time) was similar to functions fit within each individual across the four 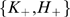 points (or four 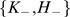 points) representing the four experimental conditions (i.e., BF, AF, BM, AM faces) (Figure 5a,b). Linear fitting to log-transformed individual data revealed a mean fit of 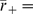
0.92±0.15 [mean±std], and 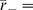
0.93±0.24 for approach and avoidance responses, respectively (Table 2; Table S2a, Supporting Information File S1 Section V). Conjunction likelihoods of observing these patterns across the cohort of healthy controls were p<7.2×10^−68^ and p<1.4×10^−162^ for approach and avoidance responses, respectively. When evaluated as a power law, the plots of individual data also related the ratios between conditions in a manner observed with the matching law [39]–[41] (e.g., AM as a referent for BF, so 
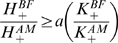
; Figure S8).

**Table 2:** Individual Data for Value and Saturation Functions, Beauty Stimuli.

Variables	Parameter	Mean±SD for Parameters
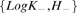	r	.93±.24
	r^2^	.92±.18
	*p* value of r	.06±.15
	*Conjunction p* value of r	1.39e-162
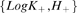	r	.92±.15
	r^2^	.86±.21
	*p* value of r	.11±.16
	*Conjunction p* value of r	7.17e-068
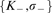	r	.95±.09
	r^2^	.90±.16
	*p* value of r	.32±.26
	*Conjunction p* value of r	<1.35e-092
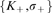	r	.97±.07
	r^2^	.95±.11
	*p* value of r	.15±.20
	*Conjunction p* value of r	<1.47e-307

Individual linear (logK, H) and quadratic (K, 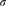) fits are listed for the subjects keypressing for beautiful and average faces. Linear and quadratic correlations were performed in each subject across the data relating to approach keypresses for the BF, AF, BM, and AM stimuli, and across the data relating to avoidance responses; subjects needed data from at least two of the experimental conditions (BF, AF, BM, and AM) to be fitted. The mean and standard deviation (SD) are listed for the correlation value, r, and for r^2^, and the likelihood value associated with r. Out of 77 subjects, 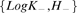 data were available for 73 subjects, 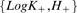 data for 46 subjects, 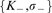 data for 65 subjects, and 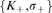 data for 50 subjects. A conjunction p value has also been computed regarding the likelihood of that number of subjects all having linear 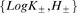 or quadratic 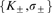 fits. The coefficient of determination, r squared, shows that 92% of the variation in 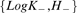, 86% of the variation in 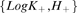, 90% of the variation in 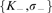, and 95% of the variation in 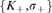 are explained by the model. Note that e-xxx denotes 10^−xxx^.

In individuals, when the relative ordering of 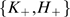 and 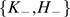 was evaluated across conditions, and the order of 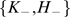 was found to not be the inverse of the order of 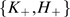, it was noted that these individuals were more distant from the central tendency of the 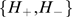 trade-off. Per condition (i.e., BF, AF, BM, AM faces), when a subject was interior to the central tendency of the 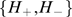 trade-off, the relative ordering of 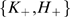 and 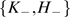 responses to that condition (e.g., AM faces) showed relatively less approach and less avoidance response than for the other conditions (e.g., BM, BF, AF faces). In contrast, when a subject was outside the central tendency of the 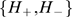 trade-off, the relative ordering of 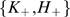 and 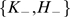 responses to that condition (e.g., BM faces) showed relatively more approach and more avoidance response than for the other conditions (e.g., BF, AM, AF faces).

The relative ordering of BF, AF, BM, AM conditions on individual 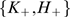 and 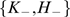 graphs varied depending on whether order was determined relative to the x axis, the y axis, or the function fit to 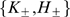 data (Figure 5c). Framing the relative ordering of experimental conditions by their log or power function fits could be determined by connecting each condition to their 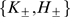 function over an absolute minimum distance. Using this approach, three types of properties (i–iii) were observed in the ordering across the BF, AF, BM, AM conditions. (i) For the 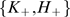 graphs, each condition showed an asymmetry of ordering such that for any two conditions 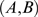, one observed *either* 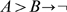 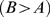 (condition “A” was greater than “B” implied the opposite was also not true, namely it was not the case that “B” was greater than “A”) *or* 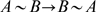 (condition “A” and “B” were similar if they were graphically superimposed). (ii) Within the 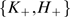 pattern, all conditions showed 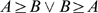 (there was a complete ordering so that either “A” was greater than or equal to “B”, or “B” was greater than or equal to “A”, so that across all four face conditions there were 16 potential orderings). (iii) Within the 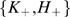 pattern, all conditions showed transitivity of ordering in that 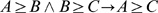 (across all face conditions, if condition “A” was greater or equal to “B”, and “B” equal to or greater than “C”, then “A” was greater or equal to “C” given their 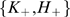 relationships). These properties of asymmetry, completeness, and transitivity observed with each 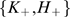 graph, were also observed for each 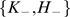 graph, and are considered properties of preference relationships [73].

#### Mean Keypress Intensity (K) and Standard Deviation (σ)

**Figure 6:**
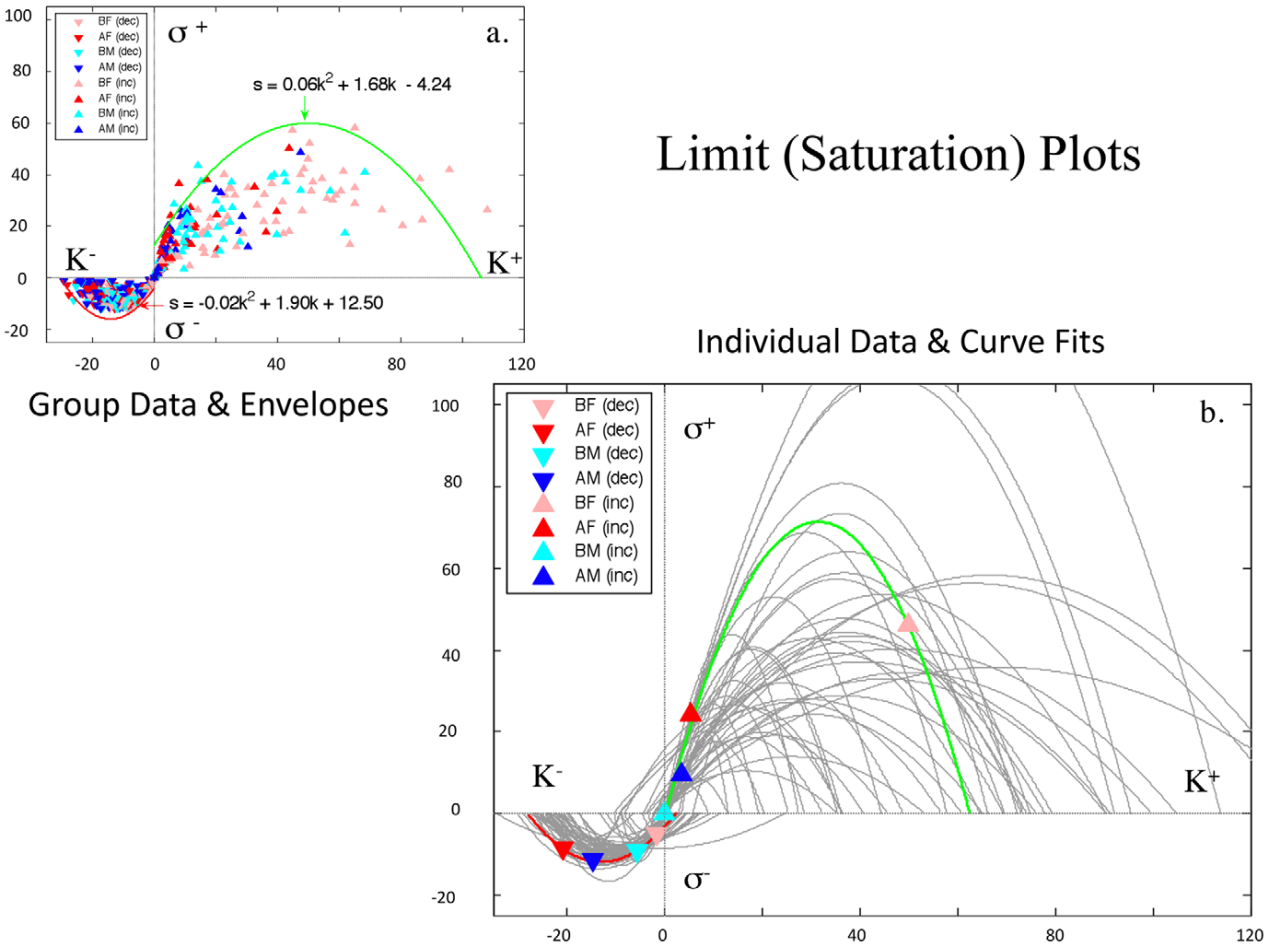
Group and Individual Saturation Plots. In (**a**), mean keypress intensity (K) is plotted against standard deviation (σ), for approach and avoidance responses to the BF, AF, BM, AM faces, in 77 control subjects. A quadratic envelope readily fits the avoidance data 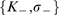, and the left side of the approach data distribution for 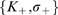. Most telling are the individual data, where quadratic fits are also observed for each of the 77 individual data sets with the BF, AF, BM, AM data (**b**). A similar mathematical structure is observed in individual graphs with the BF, AF, BM, AM faces, albeit with different fitting parameters for each of the 77 subjects. These patterns are similar to those reported for ensemble averages of mIPSCs for synaptic GABA_A_ channels by De Koninck & Mody [38].

The pattern variables SNR, CoV, and H all have relationships with the standard deviation (σ), so we also graphed 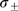 against 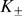, and other location measures. Graphs of group data for 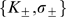 demonstrated envelopes (Figure 6a) for BF, AF, BM, AM faces that were not due to ceiling/floor effects in behavior, and could be fit with quadratic functions. Quadratic fitting of group data revealed an 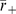 that ranged between 0.83 and 0.87, and an 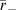 that ranged between 0.57 and 0.78 for approach and avoidance responses, respectively (Table 3; Table S3a, Supporting Information File S1 Section V). In these graphs, 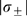 increased and then returned toward baseline, indicating a saturation relationship. The avoidance saturation envelope was more compact than the approach saturation envelope, although the general description of both was similar 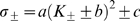 (Figure 6a; Supporting Information File S1 Section II, Pattern 3), and plots of 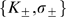 in the individual resembled the group “saturation” envelope (Figure 6b). Fitting of quadratic functions to individual data revealed a mean fit of 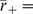
0.97±0.07, and 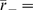
0.95±0.09 for approach and avoidance responses, respectively. Conjunction likelihoods of observing these patterns across the cohort of healthy controls were p<1.5×10^−307^ and p<1.3×10^−92^ for approach and avoidance responses, respectively (Table 2; Table S3a, Supporting Information File S1 Section V).

**Table 3:** Group Data for Saturation Function, Beauty Stimuli by Stimulus Category.

Stimulus Category	Variables	Parameter Value	
		r	Sig.
Beautiful Female	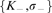	.782	<1×10^−5^
	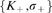	.826	<1×10^−5^
Average Female	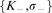	.566	<1×10^−5^
	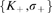	.860	<1×10^−5^
Beautiful Male	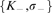	.660	<1×10^−5^
	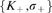	.858	<1×10^−5^
Average Male	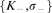	.616	<1×10^−5^
	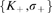	.872	<1×10^−5^

Quadratic fits for the group data from 77 subjects keypressing for beautiful and average faces. Eight correlations were performed between mean intensity data (K) and the standard deviation of the responses (sigma), stratified by the stimulus condition (BF, AF, BM, AM faces) and valence of keypress (approach, +, or avoidance −). The degrees of freedom for each quadratic correlation were df1 = 2, and df2 = 74. Results are listed for r and the related likelihood (p value or significance).

### Robustness with Noise

#### Noise Simulation

**Figure 7:**
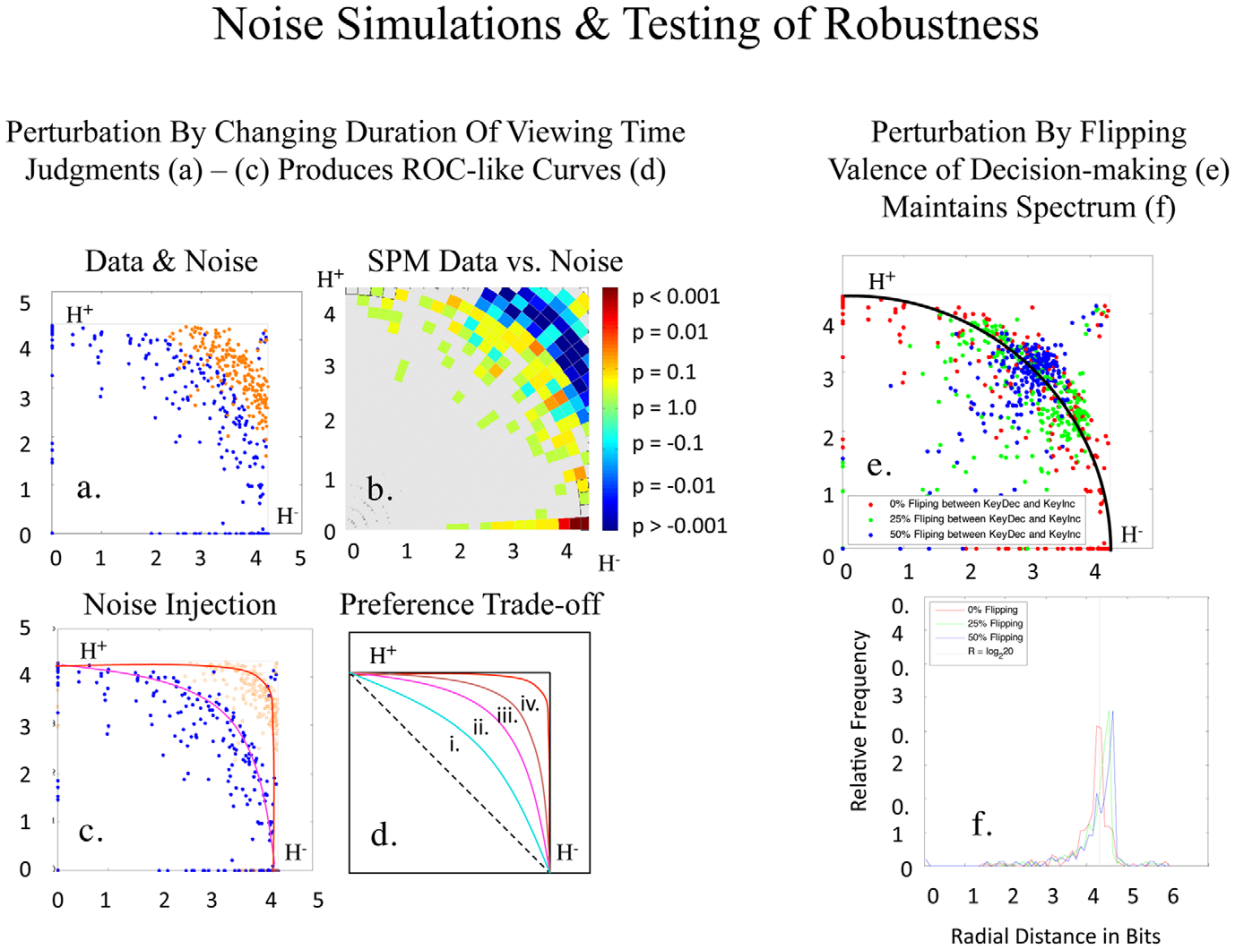
Noise Simulation and Injection for 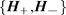. (**a**) Simulation results for variance-matched Gaussian noise (orange dots) do not mimic data from 77 controls over 4 experimental conditions (blue) (also see Figure S9). These simulation data represent alterations in the length of exposure to stimuli, and thus relate to the psychological process of judgment regarding how long to keypress for a stimulus. The minimal overlap between real data and simulated noise is underscored by statistical parametric mapping (i.e., bucket statistics (**b**)). When the Gaussian noise is injected into the real data, a new manifold is produced (orange dots), which is shifted past the manifold for the Gaussian noise (**c**). Depending on the noise distributions used for injection into experimental data, one can observe a range of central tendencies for the manifolds resulting from data plus noise, which share features with receiver operating characteristic (ROC) curves (i)–(iv) in (**d**). The cartoon in (**d**) can also be compared to Figure 3, where (i) represents the theoretical internal boundary for the trade-off manifold when subjects either keypress to approach or avoid; the central tendency of the experimental data would be (ii), while the outer border with Gaussian noise data would be (iii), and the new manifold due to injected noise would be (iv). The Pflip analysis shown in (**e**) and (**f**) allows one to assess the effects of inserting noise into the decision-making process. It specifically alters the valence or polarity of the decision-making shown by experimental subjects for their existing trace profiles in a parametric fashion (i.e., flipping 10%, 20%, 30%, 40%, 50%, etc. of the decisions from approach to avoidance, and vice versa). The graphical effect of this parametric flipping of the valence of decision-making can then be assessed by overlaying graphical representations of existing subject data with representations altered by this decision-making perturbation. In the preference trade-off graph (**e**), this flipping leads to data convergence toward the midpoint of the theoretical central tendency of the 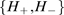 manifold as one goes from 0% flipping to 50% flipping. With 60% to 100% flipping one observes the manifold being stretched back out along its central tendency (i.e., the black line; data not shown). As one goes from 0% to 100% flipping, one effectively reverses the manifold so that it is rotated along the radius line of 45 degrees. In (**f**), we see that the radial spectra of the Pflip analysis are superimposed and similar across flipping perturbations. The 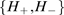 manifold is thus robust to perturbation of the decision-making.

Three noise distributions simulated in hypothetical subjects did not co-localize with graphs of 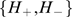 and 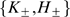 (Figure 7a; Figure S9; data not shown for 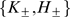), and could be segregated statistically from these graphs (Figure 7b).

#### Noise Injection/Perturbation

When variance-matched Gaussian noise was injected into 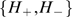, affecting judgments of preference intensity, the manifold shifted past the simulated noise (Figure 7c). Depending on the noise distribution used, one observed ROC-like curves [74] (Figure 7d), with orderly radial distributions after noise injection. In contrast, perturbations in the valence of decision-making shifted the distribution of data along the 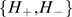 manifold, revising polar as opposed to radial distributions (Figure 7e), and minimally affecting the spectra of the manifold's radial distribution (Figure 7f). The 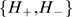 manifold thus appeared to be robust to noise perturbation/injection.

### Trial-By-Trial Response Independence

In this study, the duration for viewing each picture was determined by participants, and adjustments were not made by the experimental software to keep each trial of constant temporal length. To assure that each action was not having an effect on the following actions (e.g., whereby a subject pressing on a beautiful image for a long period of time, may press for a lesser period in a following trial), an analysis of variance was performed for the effect of preceding trial behavior (independent variable) on each subsequent trial (dependent variable) across and within subjects. Across subjects, trials of AF or BF faces before BF faces produced no effect on the duration of BF keypressing [F(1,1435) = 0.19, p = 0.67]. Trials of AF or BF faces before AF faces produced no effect on the duration of AF keypressing [F(1,1487) = 0.02, p = 0.89]. Trials of AM or BM faces before BM faces produced no effect on the duration of BM trials [F(1,1452) = 2.5, p = 0.12], and trials of AM or BM faces before AM faces produced no effect on the duration of AM trials [F(1,1470) = 0.001, p = 0.98].

Analysis within each individual subject (N = 77) was also performed to assess the effect of preceding trial viewing on subsequent trials. Within subject analysis was first performed for the number of increasing keypresses and the number of decreasing keypresses, for each of the four face categories (i.e., 8 comparisons per subject). Given 77 subjects or 616 comparisons, 29 comparisons (i.e., 4.70% of the comparisons run) produced p<0.05. If one corrected for 8 comparisons per subject, requiring a p<0.00625, only 2 of 616 comparisons met this threshold. If one evaluated within subject effects using total viewtime, only 4 comparisons were run per subject, or 308 total analyses across 77 subjects. With this analysis, 15 analyses produced p<0.05, or 4.87% of analyses run. If one corrected for multiple comparisons, requiring p<0.015, only 4 of 308 analyses met this corrected threshold.

For all analyses of trial-by-trial interactions, the number of significant effects found was less than what would be expected by chance (i.e. <5%). These results suggest that trial-by-trial viewing was, to first approximation, independent of prior behavior.

### Pattern Recurrence with Other Stimulus Sets

#### International Affective Picture System (IAPS)

**Figure 8:**
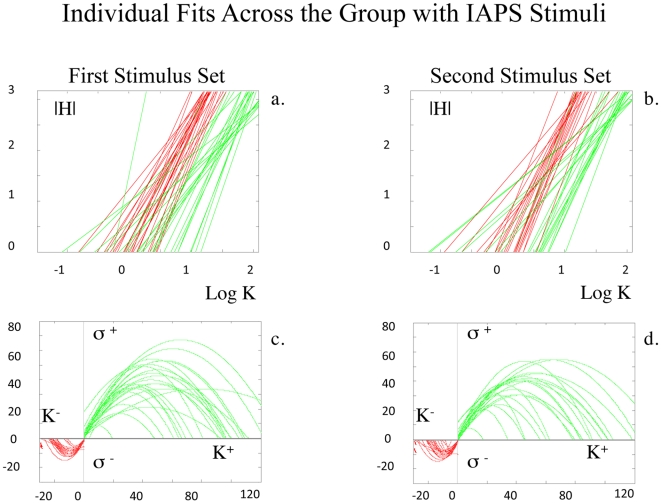
Replication with IAPS Stimuli. With transformation of the axes in (**a**) & (**b**), both the 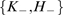 data (red linear fits) and the 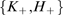 data (green linear fits) show power law scaling for the individuals in the first and second experiments with the IAPS stimuli. Saturation plots for the same individuals are shown in (**c**) & (**d**), where quadratic fits for 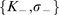 are shown in red, and for 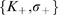 are shown in green.

**Figure 9:**
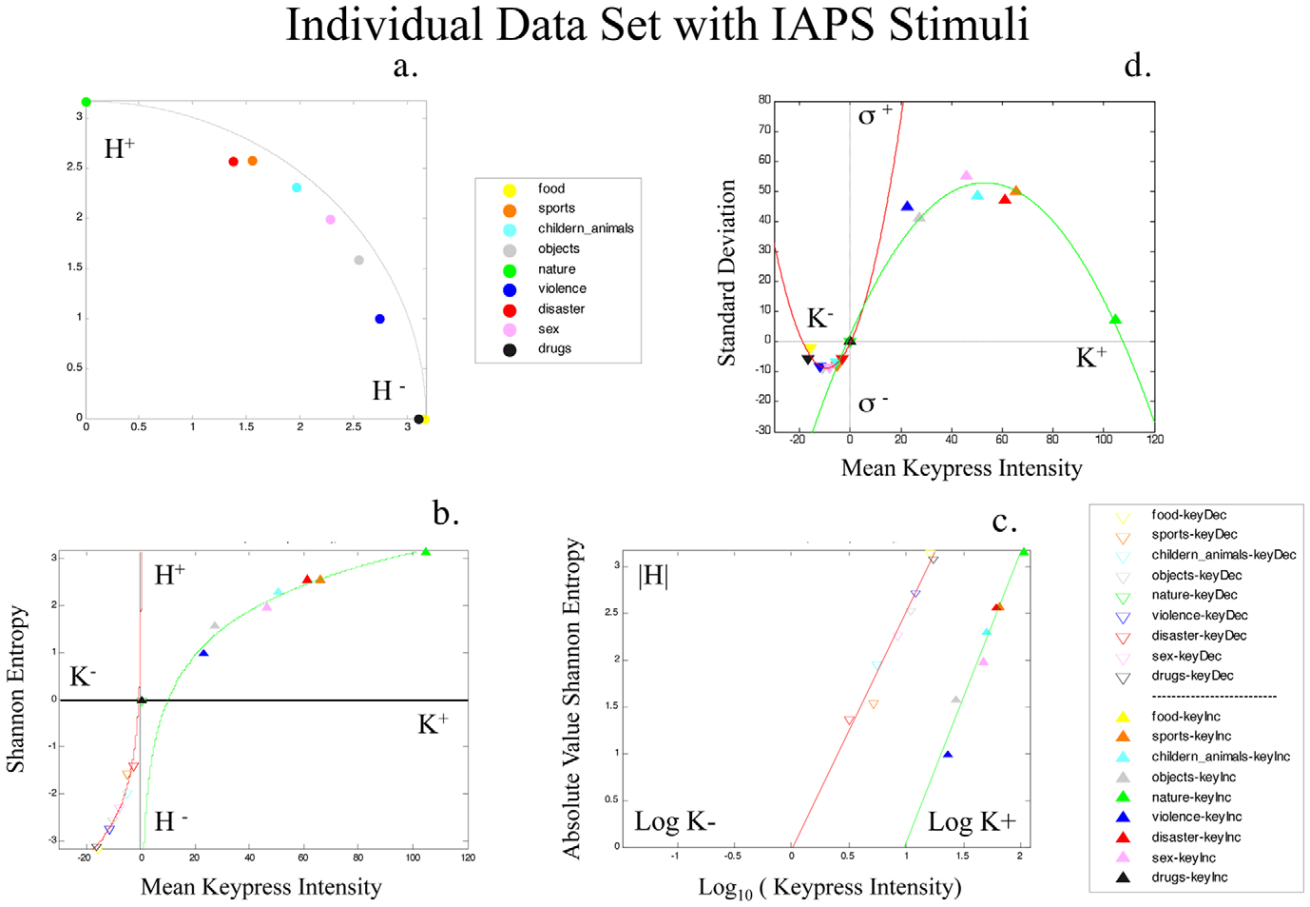
Individual Data Set from IAPS Experiment. The nine categories of stimuli used from the IAPS stimuli for these experiments are color coded, and displayed for one example subject. This subject's 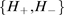 plot is shown in (**a**). Their value function, 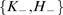 and 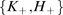, is shown in (**b**), and with log-transformation of K in (**c**). Note the tightness of the fitted functions in (**b**) and (**c**). Tight quadratic fitting is further noted for the saturation function in (**d**), for both 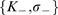 and 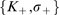. Details regarding these fits across the entire cohort of subjects undergoing testing with the IAPS stimuli, can be found in Tables 4 and 5. Note the similar sets of behavioral patterns in this figure to those shown in Figures 2–6.

In a second independent cohort of subjects, two distinct sets of IAPS pictures were tested, with 9 experimental conditions (i.e., images of children/animals, disaster, drugs, food, nature, objects, nudity, sports, violence). As with the “beauty” stimulus set, no coherent patterns were observed between location measures of positive (approach) and negative (avoidance) keypressing to IAPS stimuli. Structure (i.e., a coherent pattern) was observed in graphs between the same pattern variables for approach and avoidance keypressing used with the beauty stimuli. Structure within the 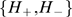 plot, 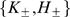 plot, and 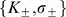 plot for the IAPS data had the same general mathematical formulation and signatures as that observed with beauty stimuli (Figure 8a–d; Table S1, Supporting Information File S1 Section V; group data for trade-off plot not shown). These coherent patterns were also clear for individual data (Figure 9a–d; Tables 4 & 5; Tables S2b & S3b, Supporting Information File S1 Section V).

**Table 4:** Individual Data for Value and Saturation Functions, IAPS, First Experiment.

Variables	Parameter	Mean±SD for Parameters
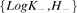	r	.96±.05
	r^2^	.93±.09
	*p* value of r	3×10^−3^±.01
	*Conjunction p* value of r	5.03e-113
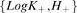	r	.94±.08
	r^2^	.90±.13
	*p* value of r	.02±.08
	*Conjunction p* value of r	<4.32e-102
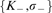	r	.90±.12
	r^2^	.82±.18
	*p* value of r	.25±.23
	*Conjunction p* value of r	2.87e-031
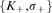	r	.95±.05
	r^2^	.90±.09
	*p* value of r	.17±.26
	*Conjunction p* value of r	<2.06e-062

Individual linear (logK, H) and quadratic (K, σ) fits are listed for the subjects keypressing for IAPS stimuli, in the first IAPS experiment. Linear and quadratic correlations were performed in each subject across the data relating to approach keypresses for the nine categories of IAPS stimuli, and across the data relating to avoidance responses; subjects needed data from at least two of the experimental conditions (children/animals, disaster, drugs, food, nature, objects, nudity, sports, violence) to be fitted. The mean and standard deviation (SD) are listed for the correlation value, r, and for r^2^, and the likelihood (p value) associated with r. Out of 33 subjects in the first experiment, 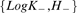 data were available for 26 subjects, 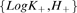 data for 25 subjects, 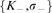 data for 26 subjects, and 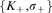 data for 25 subjects. A conjunction p value has also been computed regarding the likelihood of that number of subjects all having linear 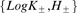 or quadratic 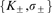 fits. The coefficient of determination, r squared, shows that 93% of the variation in 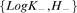, 90% of the variation in 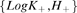, 82% of the variation in 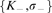, and 90% of the variation in 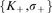 are explained by the model. Note that e-xxx denotes 10^−xxx^.

**Table 5:** Individual Data for Value and Saturation Functions, IAPS, Second Experiment.

Variables	Parameters	Mean±SD for Parameters
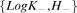	r	.94±.07
	r^2^	.89±.13
	*p* value of r	.007±.01
	*Conjunction p* value of r	6.40e-081
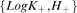	r	.94±.09
	r^2^	.88±.15
	*p* value of r	.007±.01
	*Conjunction p* value of r	2.82e-078
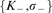	r	.91±.11
	r^2^	.85±.17
	*p* value of r	.23±.25
	*Conjunction p* value of r	2.28e-027
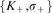	r	.92±.10
	r^2^	.86±.17
	*p* value of r	.12±.22
	*Conjunction p* value of r	8.72e-035

Individual linear (logK, H) and quadratic (K, σ) fits are listed for the subjects keypressing for IAPS stimuli, in the second IAPS experiment. Please see legend for Table 4 for definitions. Out of 31 subjects in the second experiment, 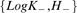 data were available for 23 subjects, 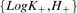 data for 21 subjects, 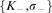 data for 23 subjects, and 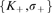 data for 21 subjects. The coefficient of determination, r squared, shows that 89% of the variation in 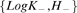, 88% of the variation in 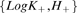, 85% of the variation in 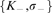, and 86% of the variation in 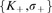 are explained by the model. Note that e-xxx denotes 10^−xxx^.

For 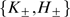 plots, linear fits to log-transformed individual data revealed a mean fit of 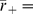
0.94±0.08 [mean±std], and 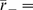
0.96±0.05 for approach and avoidance responses, for the first IAPS experiment, and a mean fit of 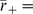
0.94±0.09, and 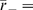
0.94±0.07 for approach and avoidance responses for the second IAPS experiment, respectively (Tables 4 & 5; Table S2b, Supporting Information File S1 Section V). Conjunction likelihoods of observing these patterns across this cohort of healthy controls were p<4.3×10^−102^ and p<5.0×10^−113^ for approach and avoidance responses with the first IAPS experiment, and p<2.8×10^−78^ and p<6.4×10^−81^ for approach and avoidance responses with the second IAPS experiment, respectively. For both the first and second IAPS experiments, 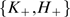 and 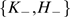 graphs showed the asymmetry, completeness, and transitivity relationships observed with the beauty data.

For the 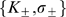 plots, quadratic fitting to individual data revealed a mean fit of 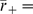
0.95±0.05, and 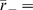
0.90±0.12 for approach and avoidance responses with the first IAPS experiment, and a mean fit of 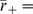
0.92±0.10, and 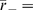
0.91±0.11 for approach and avoidance responses with the second IAPS experiment, respectively (Tables 4 & 5; Table S3b, Supporting Information File S1 Section V). Conjunction likelihoods of observing these patterns across this cohort of healthy controls were p<2.06×10^−62^ and p<2.87×10^−31^ for approach and avoidance responses with the first IAPS experiment, and p<8.72×10^−35^ and p<2.28×10^−27^ for approach and avoidance responses with the second IAPS experiment, respectively.

#### Food Stimuli

**Figure 10:**
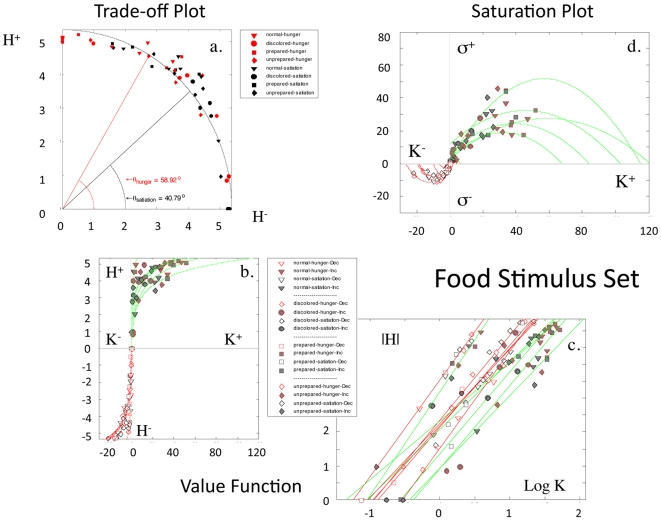
Replication with Food Stimuli. Four types of food stimuli were shown to subjects in hungry and satiated states. The order in which each state occurred was counterbalanced across subjects and separated by approximately one week. These stimuli included pictures of normal colored food, discolored food, prepared food and unprepared food. For the trade-off plot in (**a**), the center of mass of 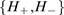 across these four stimuli differed between the hungry and satiated states, with an angular offset of 58.92° during the hunger condition, and 40.79° during the satiated condition. This difference of 18.13° quantifies the alliesthesia effect, by which homeostatic state can alter the baseline valuation of goal-objects. For these same subjects, the value function for 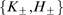 is shown in (**b**), with the signature of a power law in (**c**), and approach responses in green and avoidance responses in red. Lastly, the saturation plots for these same subjects are shown in (**d**), with quadratic fitting of approach responses in green and avoidance responses in red. Color, shape, and open/full coding of the four stimulus types, during hunger or satiation, for approach and avoidance responses, are shown with the same codes in (**b**)–(**d**). Details regarding fitting across the cohort of subjects undergoing testing with the food stimuli, can be found in Table 6. Note the similar sets of behavioral patterns in this figure to those shown in Figures 2–6, 8, and 9.

In a third independent cohort of subjects, subjects were tested twice with the same stimulus set, one week apart, in the framework of hunger or satiation. Images for the stimuli were of normal colored food, discolored food, prepared food, and unprepared food ingredients, making a total of four stimulus categories or experimental conditions. These four conditions were assessed during states of hunger and satiation, producing eight measures. As with the “beauty” stimulus set and the IAPS stimulus sets, no coherent patterns (i.e., structures) were observed between location measures of positive (approach) and negative (avoidance) keypressing to food stimuli. Structure was observed for graphs between the same pattern variables for approach and avoidance keypressing used with the beauty and the IAPS stimuli. Structure within the 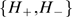 plot, 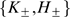 plot, and 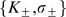 plot for the 8 food measures had the same general mathematical formulation and signatures, as that observed with beauty stimuli (Figure 10a–d). These patterns were clear for both group and individual data. Furthermore, there was a quantifiable differentiation of 18.13° in polar angle of the trade-off plot for hunger and satiation-based keypress responses (Figure 10a), quantifying the hedonic deficit state.

For 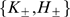 plots, linear fitting to log-transformed individual data revealed a mean fit of 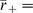
0.95±0.03 [mean±std], and 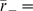
0.98±0.01 for approach and avoidance responses, respectively (Table 6; Table S2b, Supporting Information File S1 Section V). Conjunction likelihoods of observing these patterns across this cohort of healthy controls were p<8.6×10^−24^ and p<6.2×10^−28^ for approach and avoidance responses, respectively. As with experiments run with the beauty and IAPS stimuli, 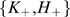 and 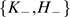 graphs from the experiments with food stimuli showed asymmetry, completeness, and transitivity relationships.

**Table 6:** Individual Data for Value and Saturation Functions, Food Stimuli.

Variables	Parameters	Mean±SD for Parameters
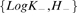	r	.98±.01
	r^2^	.96±.03
	*p* value of r	1×10^−4^±2×10^−4^
	*Conjunction p* value of r	6.23e-028
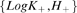	r	.95±.03
	r^2^	.91±.06
	*p* value of r	6×10^−4^±9×10^−4^
	*Conjunction p* value of r	8.64e-024
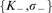	r	.91±.06
	r^2^	.82±.11
	*p* value of r	.16±.19
	*Conjunction p* value of r	5.72e-008
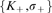	r	.91±.08
	r^2^	.83±.13
	*p* value of r	.06±.08
	*Conjunction p* value of r	3.15e-014

Individual linear (logK, H) and quadratic (K, σ) fits are listed for the subjects keypressing for food stimuli (Normal, Discolored, Prepared, and Unprepared), during states of hunger and satiation. Please see Table 4 legend for definitions. Data represents the output of six subjects. The coefficient of determination, r squared, shows that 96% of the variation in 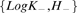, 91% of the variation in 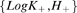, 82% of the variation in 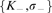, and 83% of the variation in 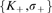 are explained by the model. Note that e-xxx denotes 10^−xxx^.

For the 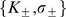 plots, quadratic fitting to individual data revealed a mean fit of 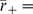
0.91±0.08, and 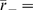
0.91±0.06 for approach and avoidance responses, respectively (Table 6; Table S3b, Supporting Information File S1 Section V). Conjunction likelihoods of observing these patterns across this cohort of healthy controls were p<3.2×10^−14^ and p<5.7×10^−8^ for approach and avoidance responses, respectively.

Given the exact same stimuli were tested one week apart, this experiment allowed a quantitative assessment of test-retest reliability. Evaluation of the relative ordering of the four food picture conditions across test sessions was performed, compared for consistency across test sessions, and tabulated across subjects. Of the four food conditions, 3.67±0.52 of them were ordered similarly between test sessions (i.e., hunger and satiation) across subjects.

## Discussion

This study found patterns connecting approach and avoidance behavior, which were recurrent across three distinct sets of stimuli (i.e., beautiful and average faces, IAPS, and food stimuli) and three groups of subjects. These patterns were specific to a small subset of behavioral variables. These patterns included (a) trade-offs that counterbalanced approach and avoidance behavior for three pattern variables (i.e., SNR, CoV, and H), (b) value functions calibrating keypress intensity (K) with each of the three pattern variables, and (c) a limit (or saturation) function connecting choice intensity (K) with its standard deviation (σ) delineating limits to approach and avoidance behavior (Figure S10; Supporting Information File S1 Section II, Patterns 1–3).

Patterns (a)–(c) were found to exhibit relationships between each other that were not always obvious to visual assessment, and necessitated simulations. For instance, most mappings inward from the 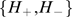 trade-off reflected both low avoidance and low approach on 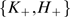 and 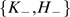 plots. Other mappings inward of the 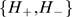 trade-off occurred for individuals who accepted the default condition more than once. Together, these observations suggested an interpretation that mappings inward of the 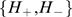 trade-off represented some degree of indifference (Figure 3). Such an interpretation does not relate to the fact there is a theoretical lower limit to the computation of 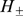 due to the number of items in a stimulus set (Pattern 4, Supporting Information File S1 Section II). In contrast to mappings inward from the 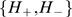 trade-off, many mappings outward from the 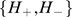 trade-off reflected both high avoidance and high approach on 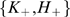 and 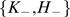 plots. A similar mapping occurred for the noise simulations and for individuals who responded with both approach and avoidance keypresses for the same stimuli. Taken together, these observations suggested an interpretation of variable amounts of psychological conflict for mappings outside the 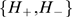 trade-off (i.e., as when you both love and hate something) (Figure 3). In addition to relationships between patterns (a) and (b), relationships were also observed between the 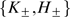 value functions (b) and 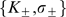 limit (saturation) functions (c), in that steeper slopes with 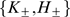 were associated with tighter saturation mappings.

These relationships across patterns (a)–(c) were relevant for generalizing the relative orderings of experimental conditions (e.g., BF, AF, BM, AM) observed within the 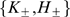 plots. Across all three experiments, the 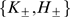 value function was observed to encode three critical features for logical constructions of preference [73], namely asymmetry, completeness, and transitivity of ordering across experimental conditions, arguing the 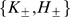 plots reflect relative preferences for individuals toward the stimuli. Given the 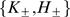 plot was shown to reflect features inherent in the 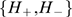 trade-off plot and the 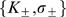 limit plot, we will use the phrase “relative preference” in subsequent text to refer to the properties of asymmetry, completeness, and transitivity observed across categories of stimuli in 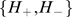, 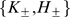 and 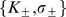 plots. Inclusion of the “relative” adjective is important given test-retest comparison with the food stimuli showed that 3.67 of 4 food conditions were similarly ordered across test sessions with altered framing (i.e., hunger vs. satiation) and were not perfectly identical. Each pattern, and what it potentially communicates about relative preference, will be discussed in the paragraphs that follow.

Trade-off plots were observed with each of the three pattern variables, yet the 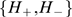 plot was the simplest in mathematical terms. The 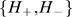 plot suggests that relative preference represents a balance between approach and avoidance choices, where bundles of approach behaviors are balanced against bundles of avoidance behaviors. Of the trade-off plots observed, the trade-off between 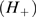 and 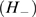 could not be simulated or produced from noise, was recurrent across all variables tested, and was robust to noise injected into the judgment and the decision-making components of the task. Shannon defined information as the uncertainty related to making a choice [67], so the preference trade-off plots between 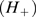 and 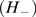 show how uncertainties regarding approach choices might be balanced against uncertainties regarding avoidance choices.

Shannon's insight has relevance for one of the value functions observed (i.e., 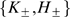 vs. 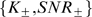 or 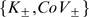), in that the 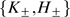 plot appears to relate preference intensity to the uncertainty associated with preference choices. These ‘intensity-uncertainty’ (i.e., 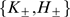) envelopes for group data (Figure 4a,c) and functions for individual graphs (Figure 5a,b) showed a relationship between approach and avoidance graphs that was similar to the positive and negative components of the value function for prospect theory (Figure 4b). The slopes for avoidance responses were steeper than the slopes for approach, which in prospect theory (Figure 4b) is interpreted as “loss aversion” [7], [12]. In prospect theory, the value function graphs a relationship between (i) the value of gains and losses in the larger economic system and (ii) subjective value or utility defined by individuals, and thus depends on a global or universal framework. In contrast, the intensity-uncertainty relationship shows a predictable pattern between two measurements within the same individual. Personal utility, the dependent variable in the value function of prospect theory [13], has been hypothesized to contain a probabilistic measure of choice [75], so that preference magnitude is connected to an estimate of the uncertainty associated with that preference. The intensity-uncertainty relationship observed in these experiments supports such a hypothesis, and places this calibration of value within a “relative” construct (please also see Figure 5c).

The intensity-uncertainty envelopes for group data and functions within individuals appeared consistent with power law scaling, producing linear correlations that were recurrent and strong. In such a framework, the plots of individual data also related the ratios of measures, which resemble the Matching Law (Figure S8). Matching describes the relative apportionment of value between reinforcers [41], [76]. Originally conceived as a linear relationship between ratios [39], [42], it has been observed to follow a power function in some cases [40]. The current data suggest that apportionment of uncertainty related to preferences (i.e., the ratio of uncertainty toward discrete experimental conditions such as BF and AM faces), was quantitatively related to the relative apportionment of preference intensity across categories of experimental stimuli. Such an interpretation would not apply to value functions that calibrated SNR or CoV to the intensity (K) of relative preference. Such an interpretation would also not apply to the strictly logarithmic interpretation of the intensity-uncertainty patterns (see Figure 4a), which ignored the presence of a logarithm in computing the Shannon entropy.

Although the 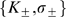 (i.e., intensity-variance) graph can be considered as derived from the intensity-uncertainty graph, the saturation relationship observed has its own implications. Humans do not act like molecules for which increased temperature (analogous to K) leads to increased variance 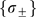. The saturation observed in the intensity-variance graph (Figures 6a,b) can be analogized to the issue of easy versus hard decision-making described by Koechlin and Hyafil [77]. Namely, decisions involving low or high preference magnitude will be easy and therefore have low variance associated with them. Goal-objects with intermediate magnitudes of preference will have high variance estimates, indicating potentially hard decisions.

These considerations regarding the 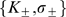 graph and decision-making may have relevance for current discussions in neuroeconomics regarding risk assessment [78]. A number of neuroimaging studies have reported neural evidence for a mean-variance approach to risk assessment [79]–[84], whereas others have provided neural evidence for an expected utility approach [30], [85]–[88]. The 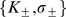 graph observed in the current study appears to be consistent with the mean-variance approach to the assessment of risky gambles [89], [90] within individuals or across groups, where risk is described by the outcome variance (i.e., the standard deviation, 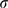). By the mean-variance approach, valuation *V* is computed by a difference between the mean transaction outcome and variance estimate: *V* 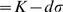, where d is the penalty imposed for risk, which increases with increasing risk aversion [78]. As illustrated in Figure S11, the 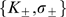 graph encodes low values of K as 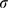 increases to a maximum, and higher values of K as 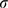 diminishes back to baseline. With K increasing as 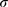 quadratically decreases, valuation *V* points to choices that are more likely to be preferred by the individual.

At the same time that the 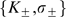 graph appears to support use of a mean-variance approach to decisions under conditions of risk, the 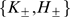 graph could be argued to support use of the expected utility approach [12], [91], [92]. For expected utility, risk aversion is thought to be represented by nonlinearity in the valuation of outcome magnitudes, as when a utility function U of an outcome K is concave, such as for prospect theory (Figure 4b). A similar concavity in the 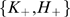 graph and convexity in the 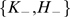 graph means that the incremental change in H+/H− is less for each change in K+ or K−. Such an analogy necessitates further research to determine to what extent the pattern of outcomes for categories of goal-objects (e.g., patterns in subplots #1–#6 in Figure 3) might serve as a representation of a utility function. Until such work is done, we can only state that the 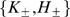 graph displays a feature akin to that of “loss aversion” in prospect theory. If future work can connect the 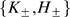 graphs with expected utility approaches to risk assessment, and the 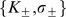 graphs with the mean-variance approach [78], such findings would provide indirect support for the dual-system theory of cognition proposed by Evans [93]. Dual-system theory, when applied to choice under uncertainty, has analogies to reflexive versus reflective learning proposed by Daw and colleagues [94] and heuristic versus logical problem solving proposed by Kahneman and Frederick [95].

The saturation data produced strong and consistent quadratic fits across all three experiments, even when there was a baseline shift in hedonic state as with the third experimental cohort. The intensity-variance graph appeared to define limits to both the intensity of preference and the range of intensities that decisions will span for individuals and groups. It should, thus, facilitate the quantification of state-based effects or adaptation in valuation [43], [45]. Indeed, each of the three functions in the third cohort of subjects showed an increase in positive preference bias during hunger relative to satiation. This can be quantified by a shift of the radial angle in the trade-off plot between the center-of-mass of data collected during the satiated condition and data collected during the hunger condition (Figure 10a). A related shift occurs along individual saturation graphs and value functions, suggesting they code alliesthesia effects.

Together, the saturation and trade-off graphs communicate limits to the range and extent of approach/avoidance, and the balance between patterns of approach and avoidance behaviors. Limitations to behavior, and the balance between distinct behaviors, are important components of what might be considered self-regulation or control of behavioral choices, allowing individuals to modify their behavior using information about changes in internal states and in the environment [96], [97]. Traditionally, self-regulation has been primarily framed by behavioral inhibition. Hence, research has focused on the capacity for inhibitory control to modify unconscious tendencies [98]–[101], which appear to be important for self-determined behavior [102], [103]. Work on inhibitory control of decision-making appears to indicate that it might increase in efficacy with recurrent use [104]. The current results contribute to this literature by identifying markers for when to potentially apply inhibition. They also suggest a variable extent of inhibition (i.e., not just a complete “no”) could be modulated by where the category of goal-object is mapped along the 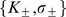 saturation plot and 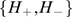 trade-off. Such considerations make analogies to control-theoretic frameworks [105]–[107] for how the variability of behavior is maintained in a narrow range, yet allowed to be tolerant of significant environmental perturbations [108], [109]. Although reminiscent of opponent process theory [110], [111], which has analogies to opponent control of color vision [112], more work is needed to evaluate how a dynamic system might target the fitting parameters of the 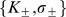 and 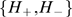 plots, or mappings on them for maintaining behavior in an optimized range.

All three patterns, the preference tradeoff, preference value function, and preference saturation function, suggested scaling between group and individual data in that they had consistent mathematical formulations across groups (as central tendencies of manifolds or boundary envelopes) and individuals (as fitted functions). In general, connections between one layer of organization and another specify the information that one has about the other [113], [114]. Statistically framed connections between scales, or graphical representations preserved across scales, directly reflect the degree to which the principles regulating organization at one scale are preserved at another [115]–[117]. This presumptive scaling between individual and group data point to a potential mechanism by which individual choice behavior in a microeconomic framework might aggregate as group behavior in a macroeconomic framework [118]. Such an interpretation is tempered by the common observation in biology [119], mathematics [120] and economics [121] of emergent behavior across layers of organization with implications that cannot readily be connected to initial conditions. Further study with a high number of experimental conditions and a very large cohort, to optimally fit individual data, would facilitate testing whether the scaling observed here between group and individual graphs can be extrapolated, as done in other studies [122], to interpret the interaction of individual preferences with the behavior of groups/markets.

Parallel research in neuroscience argues for the relevance of these findings at both the group and individual scales of measurement. Keypress intensity measures (i.e., K) have been associated (a) with reward circuitry “activation” [31], [33], [35]–[37], [50]–[52] by functional magnetic resonance imaging (fMRI) [14], and (b) with both reward circuitry activation and genetic polymorphisms connected to CREB1 [18] and BDNF [20]. Patterns similar to the 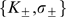 saturation plots have been produced as “variance-mean” graphs for noise analysis in electrophysiology [38], raising the prospect that the same patterns might be observed during fMRI of preference-based judgment and decision-making given the current status of knowledge regarding the basis of the blood oxygen level dependent signal [123; reviewed in 29]. Altogether, these findings argue for the “biological plausibility” [124], [125] of a number of the 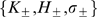 findings. Given their association with brain circuitry and genetic measures, these findings raise the question of their relevance for phenotyping psychiatric illnesses [47]–[49]. Recent work has shown that keypress intensity (K) for the four beauty face conditions was reduced in cocaine dependent subjects relative to controls [19]. The addicts also showed a restricted range in their keypress responses, and this behavior was significantly associated with reduced cortical thickness in the dorsolateral prefrontal cortex of these addicts. The restricted range in behavior is one representation of the reduced repertoire of behavior that is a defining feature of addiction in general. These findings of Makris and colleagues [19] suggest the need for further work to assess if alterations in the relationships between 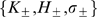 encode other quantitative features of addiction or might represent phenotypes for other psychiatric illnesses [47], [48].

Given the prospect of such applications, it is also important to consider limitations to the current work in the form of further studies needed to determine how general the findings might be. For instance, the 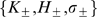 patterns appear to reflect alliesthesia effects between states of hunger and satiation, but analysis with a larger cohort is needed to quantify the consistency of such effects, and to connect them to established clinical measures of appetitive motivation [126]. Further investigation of how experimental framing might alter the ordering of items across the 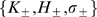 patterns, as might be expected, for instance, with sleep deprivation on carbohydrate vs. protein appetite, will be helpful to quantify the “relative” character of preference. The 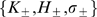 patterns were observed with a keypress procedure so it is not yet known if these patterns will be observed using data collected by other experimental methods such as ratings. Also, the length of trials was not fixed, raising the question of whether or not 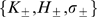 patterns would be observed if they were kept constant. The current paradigm did not show consistent dependencies between trials across subjects or within subject, but this does not rule out nonlinear effects being observed with subsequent dynamic analyses. The current paradigm used intrinsic as opposed to extrinsic goal-objects [22], [23], raising the question of whether these patterns to approach and avoidance apply to stimuli that actually meliorate a deficit state. The relevance of this last concern might be mitigated if genetic polymorphsims can be shown to directly predict variance in features of the trade-off plot, 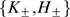 plot, or 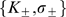 plot, or if variance in neuroimaging signals could do the same. The 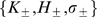 patterns were quite consistent across experiments, yet more work is needed to assess if they are mathematically closed under certain operations and thus form a function space, or might be supplemented by other relationships to form a function space. Lastly, the observation of important features from prospect theory and the matching law within the 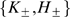 plot raises the question of a deeper relationship between the 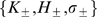 patterns, and validated constructs of reward-based judgment and decision-making. Can the 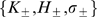 patterns be derived from prospect theory and the matching law, or vice versa? Pending studies of the issues raised above, the findings reported herein must be considered specific to the experimental paradigm used.

In summary, this study found recurrent, robust, and scalable patterns to approach and avoidance behavior. The law-like graphical patterns observed in this study are consistent with salient features from a number of established constructs regarding reward-based choice behavior. The patterns evidence the feature of loss aversion described by prospect theory [7], [12], the allocation or apportionment of preference across goal-objects described by the Matching Law [40], [76], and the limits/behavioral adaptation described by alliesthesia [43], [45]. The trade-off and value function graphs point to the idea of information processing as the discrimination among possibilities, in alignment with Shannon's initial definition of information [67], and modern frameworks for decision theory [1], [75]. At the same time, the trade-off and saturation graphs are consistent with the idea of dual processes operating to maintain the variability of behavioral output in a narrow range [108], [109]. The saturation and value function graphs raise analogies with current discussions in neuroeconomics regarding mean-variance versus expected utility approaches to assessment of risk [78]; further work is needed to assess whether the current findings contribute to such neuroeconomic discussions.

Although we focused on one set of graphical representations of the data to facilitate their integration with established findings in reward/aversion psychology, at least two other formulations are possible, albeit with more complicated parameterization (Figure S10). There may well be other formulations possible, with variable relevance to topics in the psychology of reward/aversion processing and choice behavior. How these alternate formulations scale to circuitry function is also an open question. At this time, the mean intensity of keypress behavior (K) has been associated with reward circuitry activity [14], [18], cortical thickness measures [19], and foci of genetic variability [18], [20], while variance-mean plots in electrophysiology [38] approximate the saturation plots we see with keypress behavior. These findings with (K) and (σ) variables underline the biological plausibility of the patterns described herein [124]. The apparent scaling of all 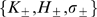 patterns between group and individual data support the neuroeconomic perspective [10], [11], [33]–[37], [124] of combining engineering [64], [127], systems modeling [60], and neuroscience [128]–[130] approaches to the study of choice behavior, and may provide a route for quantitative phenotyping of psychiatric illness [47]–[49]. Given the simplicity of the approach used to evaluate these 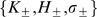 patterns, there are likely to be implications for its use within a clinical toolbox of psychological assays, and for marketing or financial applications such as agent-based macroeconomic modeling.

## Materials and Methods

### Ethics Statement

All subjects signed written informed consent prior to participation, for this study approved by the Institutional Review Board of Massachusetts General Hospital (i.e., Partners Human Research Committee, Partners Healthcare), and all experiments were conducted in accordance with the principles of the Declaration of Helsinki.

### Subjects

For the first and second cohorts, subjects were recruited by advertisement, and underwent a clinical interview with a doctoral-level clinician that included the Structured Clinical Interview for Diagnosis – Axis I (SCID-I/P) [131]. Race was determined by individual self-identification on a standardized form [132], and handedness by the Edinburgh Handedness Inventory [133]. Eligible subjects were age 18–55, without any current or lifetime DSM-IV Axis I disorder or major medical illness known to influence brain structure or function, including neurologic disease, HIV, and hepatitis C. Female subjects were studied during their mid-follicular phase based upon self-reported menstrual history with confirmation at the time of study based on an absence of progesterone surge using a urine assay. All subjects were studied at normal or corrected normal vision.

For the first cohort of subjects undergoing keypress procedures with the beauty stimuli, seventy-seven unrelated healthy subjects were recruited as participants in a multimodal imaging and genetics project, the Phenotype Genotype Project in Addiction and Mood Disorder at Massachusetts General Hospital (MGH PGP; http://pgp.mgh.harvard.edu). Subjects were adults, mean age 33.0 years (SD 11.1), mean educational history of 15.6 years (2.6), 40/77 (52%) female, and 69/77 (90%) right-handed. Ten were African American, 3 American Indian, 9 Asian, and 55 Caucasian.

For the second cohort of subjects undergoing keypress procedures with the International Affective Picture System (IAPS) stimuli [61], [62], thirty-three unrelated healthy subjects were recruited. Subjects were recruited for two experiments with the IAPS data, of which thirty-one subjects completed both experiments. For the thirty-three individuals completing the first experiment, subjects were adults, mean age 31.8 (SD 13.6), mean years of education 16.2 (±2.7 years), 21/33 (63%) females, 29/33 (87%) right handed. Four were African American, 4 Asian, and 25 Caucasian. For the thirty-one individuals completing the second experiment, subjects were adults, mean age 30.5 years (SD 13.1), mean years of education 16.2 (±2.7 years), 19/31 (61%) females, 29/31 (93%) right handed. Four were African American, 4 Asian, and 23 Caucasian.

For the third cohort of subjects undergoing keypress procedures with the food stimuli, six subjects were randomly selected from a larger cohort of fourteen subjects in an orphaned data set. This data set was collected 10 years earlier with the same keypress procedures used with the first two cohorts, but using images of food as stimuli; it was presented as a poster to the North American Association for the Study of Obesity (NAASO), October 2000, and never published. The fourteen subjects were right-handed, non-vegetarian, and free of psychiatric diagnoses (including eating disorders), neurological disease, and illicit substance dependence. Subjects were male, ages 22–40 (*M* = 27.8, *SD* = 6.1), with body-mass indices between 20.6 and 29.3 (*M* = 24.8, *SD* = 2.1). Body Mass Index (BMI) was computed as the ratio between an individual's weight and the square of his/her height (kg/m^2^). The normal range of BMI for adults is 18–25 kg/m^2^. Overweight is defined as a BMI between 25 and 30, and obesity is defined as a BMI greater than 30 [134]. No subject reported engaging in dietary restraint in order to lose weight, or smoking more than one pack of cigarettes per day. To verify that subjects exhibited normal eating behavior, we administered the Three Factor Eating Questionnaire [135], which measures three dimensions of human eating behavior: cognitive restraint, disinhibition, and perceived hunger. Subjects' mean scores (Restraint, mean 6.5, SD 4.6; Disinhibition, mean 5.9, SD 3.3; Hunger, mean 5.4, SD 3.1) were within the published normal ranges.

### Keypress and Other Experimental Procedures

#### Keypress Task

The task quantified the amount of work in units of keypress [25], [28], [136] that subjects traded for viewing time of pictures. This task used procedures and resistive function resembling those reported previously with the beauty stimuli [14], [16], [19] and with angry and other facial expressions [15], [18], [20], with the keypress procedures implemented using MatLab scripts on a PC computer.

For this procedure, categories of pictures with N pictures for each category were considered to be items in an economic bag of goods (e.g., four categories of pictures for the beauty stimulus set, with 20 identities per category). The objective was to determine, for each experimental subject, their relative approach/avoidance behavior toward the items in this bag of goods compared to the default position. Subjects were told that they would be exposed to a series of pictures that if not interfered with (i.e. no keypresses were made), would change every eight seconds (the default valuation of 6 seconds+2 second decision block; Figure 1). However, if they wanted a picture to disappear faster, they could alternate pressing the one set of keys (#3 and #4 on the button box), whereas if they wanted a picture to stay longer on the screen, they could alternate pressing other keys (#1 and #2 on the button box). Subjects thus had a choice of four potential behaviors: they could (a) approach (positive keypress), (b) avoid (negative keypress), (c) approach and avoid if they overshot or undershot a target view time, or changed their mind, or (d) do nothing about the different categories of stimuli. Keypress results reflecting viewing time for (a)–(d) were recorded as raster plots for each subject (Figure 1b). These alternatives suggest this effort-related keypress procedure (Figure 1) reflects (i) *decision-making* regarding the valence of behavior (i.e., approach, avoidance, or no action) and (ii) *judgment* regarding the amount of value that each item or face picture had relative to the default position of 6 seconds [15], [25], [29]. A “slider” was displayed left of each picture to indicate total viewing time. Subjects were informed that the task would last approximately 20 minutes, and that this length was independent of any behavioral responses to the task, as was their overall payment. The dependent measure of interest was the amount of work in units of keypress that subjects exerted in response to the different categories of stimuli (i.e., the units in keypress that the subjects traded for viewing time); work and effort exerted for experiments has become an important focus of research in effort-based decision-making over the past decade [25]–[28], [136].

To model this task, we assumed 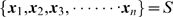, where 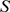 defined a set of items in a viewable itemset. The relationship of keypress effort to viewing time received, followed previous instantiations [14], [15] and was defined by the following resistive function: 
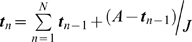
, where 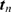 equaled the new time achieved via keypressing, 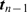 equaled the time prior to a keypress, 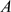 equaled Ø seconds for keypresses reducing the viewing time, and 14 seconds for keypresses increasing the viewing time, and 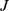 was a scaling constant set to 40. The summary of biases toward 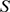 was defined by 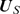 where 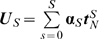 and 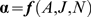 was potentially unique for each item in *S*. If the individual did nothing, then the default was: 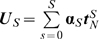 = 6 seconds×80 items = 480 seconds. With the transactions of keypress effort for changes in time, 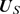 defined for each individual a set of deviations from the default position: 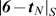.

#### Picture Stimuli for Keypress Experiments

For the three cohorts of subjects, three distinct stimulus sets were used. The first stimulus set included beautiful (models) and average (non-models) faces of both genders [i.e., four experimental conditions: beautiful female (BF), average female (AF), beautiful male (BM), and average male (AM); see Figure S1] [14], [16], [17], [19], [29]. Each of these experimental conditions consisted of either 20 male or 20 female faces. As initially developed (see acknowledgments and [14], [29]), two sets of 40 non-famous human faces were selected from print media and digitized at 600 dpi in 8-bit grayscale, spatially downsampled, and cropped to fit in an oval “window” sized 310–350 pixels wide by 470 pixels high using Photoshop 4.0 software (Adobe Systems).

The second stimulus set used images from the International Affective Picture System (IAPS) [61], [62], a well-validated stimulus set, supplemented by pictures from the Internet for only one of the nine categories of pictures (Figure S2). Pictures fell into 9 distinct categories (objects, nudes/sex, sports, disasters, food, kids/pets, nature, violence/war, and drug paraphernalia), with nine pictures per category (N = 81 items in total). For the first IAPS experiment, 5 of 9 images of drug paraphernalia came from the Internet (5 of 81 total), which were color-corrected, and reformatted for monitor viewing, with the maximum size of 1024×768 pixels. For the second experiment, 8 of 9 images of drug paraphernalia came from the Internet (8 of 81 total). These two stimulus sets of 81 pictures apiece have been referred to as “IAPS” throughout the text.

The third stimulus set used 222 photograph-quality digital pictures of food retrieved from the Internet, which were approximately 250 pixels wide and high (on average), corresponding to an area of approximately 10 degrees of visual angle on each side when viewed at a distance of 50 cm. The pictures fell into four distinct categories: (1) “Normal,” or normally-colored food items [68 pictures], (2) “Discolored,” or discolored food items [68 pictures], (3) “Prepared,” or prepared food items [43 pictures], and (4) “Unprepared,” or unprepared food ingredients [43 pictures] (Figure S3). Colors in Normal pictures were altered by PhotoShop 5.5 software (Adobe Systems) to create the Discolored picture category on an iMac DV computer [hues shifted for reds by +75, and for yellows by −110], so that this category no longer appeared natural. Prepared and Unprepared pictures were generally matched across categories of food items for the details of the food items presented, so that one presented the unprepared ingredients for a food item, and the other the prepared equivalent (e.g., a picture of a cooked steak was matched with a picture of a raw beef).

#### Other Design Procedures for Keypressing Experiments

For the beauty stimuli, the experiment was divided into two “runs”, lasting approximately 10–11 minutes apiece. The order of stimuli was randomized and reordered for each subject.

For the IAPS stimuli, each experiment was also divided into two runs lasting approximately 11 minutes apiece. Given 9 categories of images, and 9 pictures per category, pictures were presented in a counterbalanced order such that no condition followed or preceded another more than once. This produced a sequence of 41 trials for the first run, and 40 plus one trial for the second run, with the extra trial in the second run being equivalent to the last trial in the first run, placed at the beginning to maintain counterbalancing across all conditions.

For the experiments with food stimuli, each subject participated in two experimental sessions separated by 3–10 days, one in a “Hungry” state and the other in a “Satiated” state. The order was counterbalanced so that half of the subjects were in the Hungry state before the Satiated state, and vice-versa for the other half of the subjects. Although all subjects participated in both conditions, they were told in advance that their condition would be selected at random for each session, and that their condition for the second session would be independent of their condition during the first session. Each subject came to each session between 11:00 AM and 1:00 PM, having been instructed to not eat after 12:00 midnight the night before, and not at all on that day. Subjects were allowed to drink water, as well as any caffeinated beverages they would normally drink, but no other fluids. Subjects were told that they would receive a meal as part of each session. Subjects in the Hungry condition filled out the pre-session questionnaire, completed the experimental task, and were then given a meal of their choice from the hospital cafeteria; subjects in the Satiated condition were first given the meal, then filled out the pre-session questionnaire and finally completed the experiment.

### Analyses

Our general approach adapted the iterative modeling of Banks and Tran [60] to consider engineering perspectives on lawfulness [127], specifically (i) mathematical or algorithmic formulation of patterns within data, (ii) recurrency of observed patterns across discrete types of stimuli or experiments, (iii) robustness of patterns to noise, and (iv) potential scalability of observed patterns. We started with a large dataset (i.e., group data) that met stringent quality assurance criteria and interrogated it for graphical structure showing a trade-off between approach and avoidance behavior. Graphical structure focused on manifolds, boundary envelopes and fitted functions that were consistent across all the experimental conditions studied (e.g., the BF, AF, BM, and AM faces). For these analyses, we considered a manifold to be a geometric structure in the graph (i.e., a two countable Hausdorff space), which was locally homeomorphic to a 2-dimensional Euclidean space. A manifold could also have a boundary envelope or be characterized by a fitted function such as a central tendency, although a graph with an envelope or fitted function did not necessarily imply the existence of a manifold. An envelope was considered to be the boundary of the graphical region filled by mappings between location, dispersion, or pattern variables. Fitted functions were considered to be relations describable with a formula between elements in the domains of two variables [i.e., the function consisted of an ordered triple of sets (X,Y,F), where X was the domain of the function, Y the codomain, and F the set of ordered pairings between X and Y]. To determine whether an envelope or a function would be fit to a data distribution we acquired, we evaluated if the density of points at a boundary was equal to or more than the density of points elsewhere in the graph, or if the density of points fell abruptly to approximate Ø when moving away from one axis or another (and boundaries could not be the axes themselves). When consistent boundaries were observed for some data (e.g., avoidance data), but only a partial boundary was observed for data of the opposite valence (e.g., approach data), we used the mathematical format defined by the one (i.e., avoidance data) distribution, and seeded a fit with that mathematical format and the partial boundary observed for the other (i.e., approach) data. When no boundary conditions were observed (as was common with low density plots consisting of four points for one individual), function fitting was performed for the entire distribution. Please see Supporting Information File S1 Section IV, for more detail.

Where structure was found, we further assessed if variables in the trade-off were also in a relationship with other orthogonal (i.e., independent) variables regarding individual behavior. Mathematical fitting of all graphical structures and their alternate formulations was performed. Graphical structures were further tested to see if they were (a) recurrent within and across experiments, (b) distinct from any noise distributions (and potentially robust to noise), (c) scalable between group and individual data, and (d) representative of important features in the reward/aversion literature. For graphical patterns meeting (a)–(d), we then considered what novel insights these patterns provided.

#### Descriptive Statistical Measures

Keypress responses were evaluated using descriptive statistics. Location estimates included (i) mean intensity (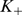 and 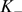 for the mean of the positive and negative keypress responses respectively), (ii) median intensity, (iii) mode of the data distribution, along with (iv) maximum and (v) minimum values in the data set (e.g., 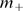 and 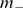 for the minimums).

Dispersion estimates for the positive and negative keypress responses focused on (iv) the standard deviation (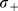 and 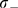), (v) the signal-to-noise ratio (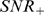 and 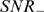), (vi) the covariance (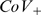 and 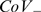), (vi) the median-absolute deviation, and (vii) the Shannon entropy (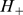 and 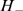; see Supporting Information File S1 Section VI). 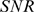 and 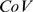 were computed as 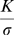 and 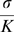, respectively. We included an entropy-based pattern variable [137] for its relevance to neuroscience and experimental psychology [64], [66]. As originally defined, it represents the *uncertainty* of making a choice [67], and thus might be of particular relevance to judgment and decision-making. The following considerations were used for computing the entropy: consider an ensemble of behavioral responses X to be a random variable x with a set of possible outcomes, 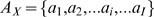, having probabilities 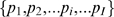, with 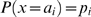, 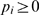 and 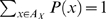. The first order entropy of this ensemble can be computed by the Shannon entropy: 
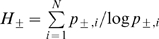
, where 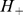 is the entropy of increasing keypresses, 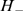 is the entropy of decreasing keypresses, 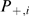 is the relative intensity of the increasing keypresses for the item (economic commodity) *i*, 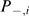 is the relative intensity of the decreasing keypresses for the item (economic commodity) *i*, and N is the number of the alternatives. For cases where subjects made no keypress responses, but accepted the default condition for all items in the itemset for a condition, we defined H = 0. H would also equal 0 in the theoretical context that the number of items in a category of economic commodity, 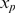, was decreased to 1, so that the individual could not, by definition, have any ambiguity of choice (Pattern 4, Supporting Information File S1 Section II). This information theoretic approach [64], [66], [67], [137]–[143] is grounded in the classical theory of entropy, but does not necessitate the temporal evolution of an ensemble. See the end of Supporting Information File S1 Section VI for an example computation.

#### Testing for recurrent trade-offs in approach/avoidance keypressing

Relationships were assessed between location estimates of keypress responses (e.g., 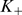 and 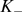), between dispersion measures (e.g., 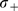 and 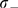), and between pattern variables (e.g., 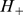 and 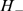). This evaluation sought to determine (a) if limit conditions were observed for any of the variables (e.g., floor/ceiling effects such as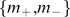 = 0), (b) if graphical structure was observed in the form of a manifold, envelope, or function, which was consistent or recurrent across all the four experimental conditions (e.g., BF, AF, BM, AM), (c) if any graphical structure balanced positive and negative keypress responses in an opponent/trade-off manner, (d) if any structure observed was mathematically simple, and (e) if the measures/estimates comprising this structure could be combined with other measures/estimates to produce structures of relevance to prospect theory [7], [12], [13], or other reward-based theories [41]. Patterns observed with individual data that were not associated with patterns at the level of group data were noted but not analyzed further. By definition, such individual patterns would not be scalable to group data.

#### Assessing other recurrent relationships between location/dispersion measures

We next evaluated whether structure observed in the graph of one location measure or dispersion estimate was related to structure observed in graphs of other measures/estimates (i.e., graphical interactions such as 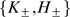 between orthogonal or independent variables). If other manifolds, envelopes, or functions were apparent that could be explicitly simulated/fitted (see Supporting Information File S1 Section IV), we then explored relationships between measures/estimates found in these new structures, and the measures/estimates that might have a relationship with them (e.g., exploring 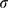, given its relationship with the pattern variables). Follow-up analyses also identified parameters influencing graphical extents for any structure by simulating constraints to choice behavior in the experimental task, to assure apparent structures were not mathematically trivial (e.g., Supporting Information File S1 Section III). We further identified critical features within and between graphical structures.

For the full set of graphs demonstrating a manifold, envelope, or function, which were consistent across all conditions (e.g., BF, AF, BM, AM faces), we then applied two procedures to confirm recurrence across experimental conditions and to test for power law scaling. The first procedure involved radial and angular re-sampling of the observed structural relationship between variables to assess the frequency spectra of their distributions and potential Gaussian, log-logistic, or t location-scale fit (Supporting Information File S1 Section I), allowing testing of whether or not they had similar central tendencies for BF, AF, BM, AM conditions. The second procedure further assessed if power law scaling was observed between variables (i) by appropriate log transformations of axes, (ii) by mathematical evaluation of a power function fit to the observed graphical structure, and (iii) by assessing if a scaling of the independent variable by a constant caused a proportionate scaling of the function itself (i.e., if given 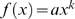, that 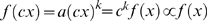) [71], [72], [144], [145]. Power law scaling would argue that the observed structure was due to self-organizational processes [68]–[70], and facilitate interpretation in light of other power functions such as the Weber-Fechner-Stevens Law [146]–[150] and value function of prospect theory [7], [12], [13].

If recurrent structure was confirmed across experimental conditions, or power law scaling observed, we lastly assessed similarities in graphical structure between (i) group data for one condition, and (ii) individual graphs involving multiple conditions to assess whether these patterns might be scalable. Evaluation of graphical similarity entailed determining if graphs from each individual had a similar mathematical form (albeit with different parameter fits) to the manifold, envelope, or function from group data.

#### Analysis of approach and avoidance in regards to preference

For this process, individual data was first reduced to rank orderings, and then evaluated for three properties relevant for preference judgments, namely rank order “asymmetry”, “completeness”, and “transitivity” across conditions (defined below) [73]. Rank ordering of experimental conditions was performed for the beauty stimuli, IAPS, and food stimuli for each subject, by connecting each experimental condition to the power function fit of their 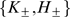 data over an absolute minimum distance (see cartoon in Figure 5c). This data was tabulated for each subject, with orderings along 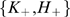 and along 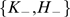 in different rows.

“Asymmetry” across experimental conditions was defined as such: for any two conditions 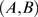, one observed either 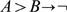 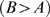 or 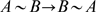. Namely, for any two experimental conditions A or B (e.g., BF and AF), condition A was greater than B implied the opposite was also not true, namely it was not the case that B was greater than A, or condition A and B were similar in that they were graphically superimposed. To test for this across each of the three data sets, we evaluated asymmetry across all potential pairings of experimental conditions within each stimulus set and cohort.

“Completeness” across experimental conditions was defined by the observation that every pairing of conditions showed 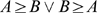. Namely, there was no experimental condition that could not be ordered relative to all of the other experimental conditions, so that either A was greater than or equal to B, or B was greater than or equal to A. This evaluation assessed completeness across four conditions for the beauty stimuli, 9 conditions for the IAPS, and 4 conditions (in two experiments) for the food stimuli.

“Transitivity” across conditions was defined as 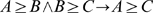. Namely, if condition A was greater or equal to B, and B equal to or greater than C, then A was greater or equal to C given their 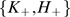 relationships. To test for this property across each of the three data sets, we evaluated asymmetry across all possible combinations of three conditions in each stimulus set, and tested if these asymmetries were transitive.

#### Analysis of graphical robustness

For graphs showing structure across subjects with the beauty stimuli (e.g., 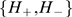, 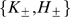), we performed two sets of control analyses: noise simulations and noise perturbations.

Noise Simulations: First, we compared group data with simulations from hypothetical subjects. These simulations were performed with each 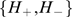 and 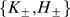 plot to determine that the patterns were not mathematically trivial in that (a) the behavioral and noise sources could be segregated, (b) the behavioral effect was not a random effect, and (c) the observed structure did not simply reflect the analytical procedures utilized. Three simulations were performed with the following noise parameterization: (i) mean-matched uniform random noise, (ii) range-matched uniform random noise, and (iii) variance-matched Gaussian noise that maximized the entropy of the response profile distribution. For all simulations, we assumed the existence of a hypothetical subject for each real experimental subject (i.e., a one-to-one match between the hypothetical subject and the experimental subject whom we studied), who was asked to keypress without any visual stimuli, over a time interval that was set so that their keypress behavior was the same as that of the experimental subject on the macroscopic level [i.e., mean, range, or variance is matched with the experimental subject], but showed random microscopic behavior within one of the macroscopic constraints. For both the mean-matched uniform noise simulation and the range-matched uniform noise simulation, the random microscopic behavior was defined as having a linearly uniform spectrum density. For the mean-matched uniform noise simulation, this meant that each hypothetical subject produced keypress results within the range of the 95% confidence interval of their matched real experimental subject; for some subjects, the lower limit of the confidence interval would be below zero, in which case the theoretical values below zero would be considered to be zero. For the range-matched uniform noise simulation, the assumption of a linearly uniform spectrum density meant that each hypothetical subject produced a keypress range that was the same as their matched real subject, with a distribution that was uniform across this range. For the variance-matched Gaussian noise, the random microscopic behavior was defined as having the same spectral density as a Gaussian, i.e., the entropy of the distribution was maximized with the given variance constraint. As with other noise distributions, some subjects evidenced a confidence interval with al lower limit below zero, in which case the theoretical values below zero would be considered to be zero. The collected behaviors of the hypothetical subjects for each parameterization were compared with those of the experimental subjects. All of the simulations were performed using random noise generation by built-in MatLab functions: the uniform noise generator or the Gaussian noise generator.

To quantify differences between experimental data versus simulated data, we applied a variation of bucket statistics used in statistical parametric mapping of neuroimaging data [151]. We applied this technique to the preference trade-off plots, and pixilated these graphs in the radial and polar dimensions. The incidence of real and hypothetical subject presence in each bucket or pixel was compared to a Gaussian distribution, in a t-statistic analysis. The t-value was converted into a pseudocolor map on the preference trade-off plot as is done commonly with neuroimaging data (Figure 7b), quantifying the segregation of experimental data from simulated data.

Noise Perturbations: Following quantifiable dissociation of real from hypothetical data, we pursued analyses assessing noise effects on the robustness of the observed graphical structures, using two approaches: injecting noise into the judgment of preference intensity and perturbing the valence (i.e., flipping a percentage of responses – “Pflip”) of decision-making data. These control analyses evaluated whether or not the general structures observed across subjects were maintained despite introduction of noise into judgment and decision-making.

The first approach to noise perturbation involved injecting noise into the existing data and assessing its graphical effects. This was performed by adding together keypress response profiles from (i) the real data and (ii) the hypothetical data (i.e., noise simulation data described above), on a one-to-one basis to decrease the contrast in the profiles between stimuli items. For this addition of hypothetical data, we added simulated data to the existing keypress response profiles with the same mean and variance as the existing data. As an extension of this process, we also added together data from two existing experimental conditions (i.e., the BF+AF data or the BM+AM data), and evaluated the graphical outcomes.

A second approach sought to undermine the polarity of the decision-making by experimental subjects through parametrically altering the percentage of traces for which the polarity was reversed in the existing response profiles and assessing the graphical effects. This perturbation flipped the valance in decision-making from the positive keypress to negative keypress and vice-versa (i.e., flipping the approach/avoidance status of response traces) with a probability of “Pflip” or P_flip_. For these processes, the random number generator used a fixed random number seed.

These two approaches to simulation allowed us to assess graphical robustness of each structure to noise, in that the graphical representation of existing subject data could be overlain with representations altered by (i) injected noise (or addition of existing keypress profiles across experimental conditions), or (ii) decision-making perturbations. Changes to the fitting parameters of any manifold, envelope, or function could then be readily determined, or dissipation of the structure confirmed.

#### Analysis of trial-by-trial response independence

This analysis sought to determine if approach or avoidance behaviors for beauty stimuli had an effect on the actions that followed. This was initially performed across subjects for each of the four experimental conditions using F tests, and then done within individuals using an analysis of variance. Given experimental conditions were segregated by gender to assure gender effects did not skew responses (see Figure 1b), F tests across subjects assessed if preceding AF and BF behavior affected subsequent BF responses, and separately, subsequent AF responses. Similarly, F tests across subjects assessed if preceding AM and BM behavior affected subsequent BM responses, or, separately, subsequent AM responses. For analyses within individuals, ANOVAs were performed for each subject with pre-condition behavior as the independent variable and post-viewing time as the dependent variable. This was performed initially for decreasing keypress behavior and increasing keypress behavior separately, and then repeated with total viewtime. Hence ANOVAs were run for four experimental conditions×increasing/decreasing keypresses×77 subjects, or 616 comparisons. This was followed by ANOVAs for four experimental conditions×total viewtime×77 subjects, or 308 comparisons. The percentage of p-values less than 0.05 were then computed to determine if they were in the range of 5% of the total number of comparisons run, or what might be expected by chance.

#### Analysis of test-retest reliability

To test for consistency of responses across test session, individual data from the experiment with food stimuli was first reduced to rank orderings, and then compared across the two test sessions performed 3–10 days apart. Rank ordering of experimental conditions was performed for each subject, by connecting each experimental condition to the power function fit of their 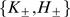 data over an absolute minimum distance (see Figure 5c). This data was tabulated separately for orderings along 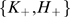 and along 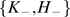 for each subject. In the evaluation of consistency, given any change in the rank order for an experimental condition could shift each of the other orderings by one position, we considered ordering preserved if it was plus or minus one position. Consider, for example, the relative orderings along 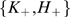 as follows: normal colored food (4,4), discolored food (2,3), prepared food (3,1), and unprepared food (1,2). In this scenario, the relative order of the normal colored food, discolored food, and unprepared food to each other was preserved, and thus this ordering was considered consistent for three experimental conditions. These results were then summarized with descriptive statistics.

## Supporting Information

File S1 Supporting information (Sections I–VI) for the main text, with mathematical description of findings, computer code for simulations, and further information about methods.
(0.43 MB DOC)

Figure S1 Examples of Beauty Stimuli. A sample of the four picture types used for the beauty stimuli (from left to right): beautiful female, average female, beautiful male and average male. Each of these experimental conditions or categories of picture consisted of either 20 male or 20 female faces. Since initial development (see acknowledgments and [14], [29]), these stimuli have been used in a number of studies [16], [17], [19].
(1.06 MB TIF)

Figure S2 Representative pictures from the International Affective Picture System (IAPS) [61], [62]. Images used came from nine distinct categories of picture content: objects, nudes/sex, sports, disasters, food, kids/pets, nature, violence/war, and drug paraphernalia. Each category contained 9 pictures. Please see Methods, *Picture Stimuli for Keypress Experiments*, for further information and commentary.
(3.01 MB TIF)

Figure S3 Examples of Food Stimuli. One example of items from each category of food stimuli: **(a)** Normally colored food item; **(b)** Discolored food item; **(c)** Prepared food item; **(d)** Unprepared food item.
(2.07 MB TIF)

Figure S4Examples of Trade-off plots Using Pattern-variables. Three functionally similar types of preference trade-off graphs are displayed for H, SNR, and CoV estimates. In **(a)**, a graph is displayed of the Shannon entropy for increasing keypress responses (y axis) versus the entropy for decreasing keypress responses (x axis) for responses to BF, AF, AM, AM faces in 77 healthy control subjects. For the same set of experimental subjects, we show in **(b)** a graph for the 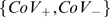 manifold, and in **(c)** a graph for 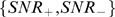, which represents a boundary envelope.(0.25 MB TIF)

Figure S5 Radial Distributions from Trade-off Plots. Each graph represents the data from 77 healthy controls, for one experimental condition (i.e., BF, AF, BM, AM faces), with three types of fitting of the radial distribution from the trade-off plot of that experimental condition. Radial sampling of the preference trade-off graphs for these four experimental conditions were tabulated using bins of 0.2 bits (in gray-tone lines off the x-axis). Bin height reflected the normalized number of data points across 77 subjects. Fitting through three methods (see Supporting Information File S1 Section I) was performed, so that each of the resulting curves contains the same area or number of samples. Qualitatively, the best fit is observed with the t location-scale distribution.
(0.46 MB TIF)

Figure S6Trade-off Plots for Total Viewtime Versus Keypress Number. The resistive function used to translate keypress effort into viewing time theoretically might influence the form of the preference trade-off, the value function or the saturation function. To rule this out, we analyzed total viewtime data (symbolized by T(K+) or T(K−) for viewtimes resulting from pressing the positive keys or the negative keys respectively), to determine if the same set of patterns was observed with group data, or whether there were discrete functions with individual data. The resulting graphs of the preference trade-off, value function, and saturation function exhibited the same patterns whether or not using keypress number or viewtime data. In this figure, the 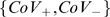 manifold is shown for keypress data **(a)** and for viewtime data **(b)**. To further support the observation shown in Figure S4, the same comparison of keypress versus total viewtime data (again using the symbolization of T(K+) or T(K−) for viewtimes resulting from pressing the positive keys or the negative keys respectively) is shown using SNR estimates. The 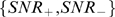 plot is shown for keypress number **(c)** and total veiwtime **(d)**. This observation further supports the potential for these analyses to be used for other frequency data besides that acquired using keypress procedures.(0.32 MB TIF)

Figure S7Examples of Value Function Graphs Using Pattern-variables. Three types of value function graphs are displayed for H, SNR, and CoV estimates. In **(a)**, graphs of 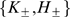 are shown in red for the negative (avoidance) keypress and green for the positive (approach) keypress for 77 healthy controls, with no color-coding between BF, AF, BM, AM stimulus conditions. The approach and avoidance keypress data are displayed on the same axes to illustrate the difference in curvature between approach and avoidance responses, which approximates the observation of “loss aversion” described in prospect theory. Similar differences between approach and avoidance slopes are also observed for value functions using SNR and CoV estimates. The boundary envelopes for 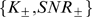 graphs **(b)**, and for 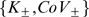 graphs **(c)** are also shown for BF, AF, BM, AM faces in 77 healthy control subjects. In **(d)**, a cartoon of the differences in boundary envelopes observed across value functions with H, SNR, and CoV estimates is illustrated. Note that a similar graphical structure to that observed with the 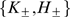 plot is also observed with a very different psychological phenomenon, namely the Weber-Fechner-Stevens Law in sensory psychophysics [146]–[150], underscoring the pervasiveness of power functions in nature [68]–[72].(0.37 MB TIF)

Figure S8Comparing the Value Function with the Matching Law. The matching law as described by Herrnstein [39] was initially approximated by ratios. Later work by Baum [40] suggested that matching could be better described by a power function, although modern research regarding matching works elegantly with the initial formulation of Herrnstein [39]. These issues are of interest given the observation of power law scaling with the 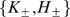 value function, which allows ratios to be represented with the same mathematical structure (equation at bottom) for individual data (two plots on right).(0.35 MB TIF)

Figure S9 Noise Simulations. Three noise simulations were run for each of the four experimental conditions, and combined in the illustrated graphs (real subject data with filled circles, and hypothetical subject data with x's or open circles). These three simulations include: **(a)** mean-matched uniform random noise, **(b)** range-matched uniform random noise, and **(c)** variance-matched Gaussian noise. Procedures for these simulations are described in the main text methods section. Note that across these three graphs, no simulation duplicates the human experimental data, or a subset thereof.
(0.30 MB TIF)

Figure S10Alternate Sets of Patterns Characterizing Approach and Avoidance. There appear to be at least three alternate formulations of the relationships organizing relative preference in humans. These three formulations are illustrated schematically in three columns of graphs, with a trade-off relationship on top of each, a value function in the middle, and a saturation function on the bottom. With group data, the trade-off relationships represent manifolds for the 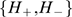 plot **(a)** and 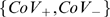 plot **(c)**, and a boundary envelope for the 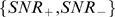 plot **(b)**. The central tendency of the 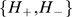 plot has a similar mathematical form to the graphs of individual data across experimental conditions tested, although there can be significant variability across individuals. For all of the value functions assessed, group data reveals an envelope for the 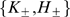, 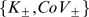, and 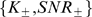 plots. In individuals, 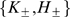 plots reveal striking functional fits. Lastly, one can associate the 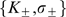 plot with each of the graphs produced using the three pattern variables.(0.39 MB TIF)

Figure S11

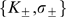
 Plot and Mean-Variance Model of Choice. The 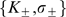 plot may have relevance for mean-variance approaches to decision making under risk. As described by D'Acremont and Bossaerts [78], the mean-variance approach describes risk by the outcome variance (i.e., standard deviation, 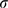), and computes a valuation *V* by the difference between the mean transaction outcome and variance estimate: *V* 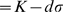, where d is the penalty imposed for risk. As d increases, the individual shows increasing risk aversion. In the quadratic fitting of 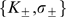, the computation of *V* is not likely to show that the individual prefers mappings on the 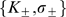 plot until after 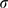 has reached a maximum and is decreasing (while K continues to increase). This might not be necessary if d is quite low, in which case one could imagine preferred choices being represented on the 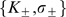 plot by mappings with low 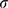, and either high or low K. Given the 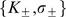 plot involves both approach (positive) and avoidance (negative) components, one might also imagine adapting the mean-variance framework to include both components in the valuation computation (e.g., so that 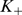, 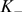, 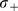, and 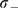 are all incorporated in the computation).(0.33 MB TIF)
