# Supplementary material for: Recurrent, Robust and Scalable Patterns Underlie Human Approach and Avoidance
Source: PLoS One. 2010 May 26;5(5):e10613. doi: 10.1371/journal.pone.0010613 (PMC2879576; doi:10.1371/journal.pone.0010613)
Supplement: File S1 — Supporting information (Sections I–VI) for the main text, with mathematical description of findings, computer code for simulations, and further information about methods. (0.43 MB DOC) [file pone.0010613.s001.doc]

**Supporting Information (Sections I – VI)**

*Recurrent, robust and scalable patterns underlie human approach and avoidance*

*Kim et al.*

**Supporting Information Section I:** Fitting of Spectra from Radial Sampling of HH

This process started by collecting the radial distribution of the graphs for each experimental condition (i.e., BF, AF, BM, AM faces). This data for 77 healthy control subjects is shown in the four graphs in Figure S5. In each of these four graphs, the x-axis relates the distance in bits from the origin, and bins shown in gray line represent 0.2 bits along the x-axis. The y-axis represents the number of data points per bin (the normalized distribution density, or the number of subjects within the corresponding bin). For each of the three fitting curves, fits are normalized (on the same scale) because they are fitted on the same data (hence the area under any fitting curve will yield the same number of the data points). Three distributions were fit to this data: the normal distribution, logistic distribution, and t location-scale distribution. The functions used for this fitting are listed below. Qualitatively, the t location-scale distribution best fits the data shown in all four graphs in Figure S5, collected across 77 healthy controls using four beauty face conditions.

<Normal Distribution>

The normal distribution is a two parameter family of curves. The first parameter, µ, is the mean. The second, σ, is the standard deviation. The standard normal distribution (written Φ(x)) sets µ to 0 and σ to 1,

<Logistic Distribution>

The logistic distribution has the density function with location parameter µ and scale parameter σ > 0, for all real x,

<t Location-Scale Distribution>

The t location-scale distribution has the density function with location parameter µ, scale parameter σ > 0, and shape parameter ν > 0. If x has a t location-scale distribution, with parameters µ, σ, and ν, then has a Student's t distribution with ν degrees of freedom, and

**Supporting Information Section II:** A Synopsis of Relative Preference Patterns

It is important to note that there are at least three potential sets of relative preference patterns. What is described in synopsis form below is one set of these patterns, related to the variables, with a limit condition.

Pattern 1. Preference Uncertainty Trade-off.

The trade-off is observed as a manifold, which represents a balance between the heterogeneity/irregularity of approach and avoidance behaviors. We graph this manifold using:

, where $H_{+}$ is the entropy of increasing keypresses, $H_{-}$ is the entropy of decreasing keypresses, $P_{+,i}$is the relative intensity of the increasing keypresses for the item (or economic commodity) *i*, $P_{-,i}$ is the relative intensity of the decreasing keypresses for the item (or economic commodity) *i*, and N is the number of the alternatives. Given an ensemble $S$ with $N$ items and M subjects making transactions recording their preferences for those items, for each subject there exists a random variable $r_{x}$ characterizing the radial distance from the origin for an graphing

where “” means that:

, as

and the probability that

when , , and

, where

Note: This one-simplex manifold characterizes closed ensembles of N.

Pattern 2. Preference Value Function or boundary envelope: calibrating response intensity and uncertainty.

As a power function, this pattern has the form: , where $H_{+}$is the entropy of increasing keypresses, $H_{-}$ is the entropy of decreasing keypresses, $K_{+}$ is the mean intensity of the increasing keypresses, and $K_{-}$ is the mean intensity of the decreasing keypresses, and a – d are fitting parameters.

If one assumes a logarithmic relationship, then one can have an alternate form for this function: , with a – c as fitting parameters.

Pattern 3. Preference Saturation Function or boundary envelope: mapping of response intensity and standard deviation.

The relationship can be modeled as: , where $s_{+}$is the standard deviation for increasing keypresses, $s_{-}$ is the standard deviation for decreasing keypresses, $K_{+}$ is the mean intensity of the increasing keypresses, and $K_{-}$ is the mean intensity of the decreasing keypresses, and a – c are fitting parameters.

Pattern 4. Lower limit of response irregularity.

As the number of items in a category of economic commodity, $x_{p}$, decreases to 1, the $H_{\pm}$ approaches 0 (meaning there is no ambiguity of choice). Please also see discussion in Supporting Information Section VI.

**Supporting Information Section III:** Behavior Simulations

Multiple analyses sought to identify parameters influencing the graphical extents of patterns observed in keypress behavior. For example, one simulation assessed if limiting keypress responses to only approach or avoidance (i.e., yoking the responses) would reproduce the preference trade-off graph. It did not, but did approximate an internal distribution of the HH manifold, suggesting a cognitive model for subjects along this internal edge (Figure 3). The Matlab code for yoked behavior (when responses are limited to only approach or avoidance) is presented below:

%%% MatLab code for yoked simulation %%%

N = 20;

pN = 1/N;

fprintf('    t-    H-    H+     r\n');

for t1=1:19 % 1 <= t1 <= N

    t2 = t1+N;  % N < t2 <= 2*N

    % Entropy on t1 side

    i1 = floor(t1);

    p1 = i1/N;

    H1 = -(p1*log2(p1) + (N - i1)*pN*log2(pN));

    % Entropy on t2 side

    i2 = 2*N - ceil(t2);

    p2 = i2/N;

    H2 = -(p2*log2(p2) + (N - i2)*pN*log2(pN));

    r = sqrt(H1*H1 + H2*H2);

    fprintf('%6d%6.2f%6.2f%6.2f\n',t1, H1, H2, r);

    x(t1) = H1;

    y(t1) = H2;

end

**Supporting Information Section IV:** Value Function Envelopes

Envelope fitting was performed whenever the density of points at a boundary was equal to or more than the density of points elsewhere in the graph, or the density of points fell abruptly to approximate Ø when moving away from one axis or another (and boundaries could not be the axes themselves). When no such conditions were observed (as was common with low density plots consisting of four points for one individual), function fitting was performed for the entire distribution (using MATLAB built-in functions). Given the scatter of data for graphs of with the BF, AF, BM, AM conditions, envelope fitting defined the boundary of the graphical region filled by data from each face category (i.e., experimental condition) across subjects. Simulations revealed envelopes that could be formulated in either power or logarithmic format. In a power format, the envelope has the form: , where $H_{+}$is the entropy of increasing keypresses, $H_{-}$ is the entropy of decreasing keypresses, $K_{+}$ is the mean intensity of the increasing keypresses, and $K_{-}$ is the mean intensity of the decreasing keypresses. The envelope for and has an upper saturation when $H=H_{\text{max}}$ at , and a lower saturation where $H_{\pm}=f$ of $K=(-c)^{1/b}-a$.

For a logarithmic relationship between $K_{\pm}$ and $H_{\pm}$, the envelope has the form: . In this case, the envelope for and has an upper saturation at , and a lower saturation where $H_{\pm}=f$ of . The scaling factors *a, b, c* represent the vertical displacement, horizontal displacement, and curvature, respectively.

For fitting linear envelopes during power law scaling (i.e., log transformation of axes) in subjects, we utilized an algorithm with iterative alterations of a tolerance parameter until we observed minimal changes in a ratio of slopes for over [71,72,144,145]. This algorithm has four general steps, and the final simulation results used a tolerance of 0.1. The Matlab script is listed below:

% Algorithm of the envelope simulation -------------------------------------

% S1. Pick a point (pt1) that should be on the desired envelope line.

% S2. Find a line (L1) that goes through pt1 and another point pt2,

% such that L1 has no outlier.

% S3. Find a line (L2) that goes through pt1 and another point pt3,

% such that L2 has no outlier except for pt1.

% S4. If the difference between the x-intercept of L2 and that of L1 is greater

% than the preset tolerance, then delete the outlier point (i.e., pt2)

% and redo from S2.

%

% Input of the function is a set of 2D points and the various options.

% In the simulation, only the right-side envelops were obtained.

%

%

function [slope, xIntercept, pt1] = envelop_simulation(x, y, tolerance,...

xintercept_resolution, xintercept_start, xintercept_end, leftright, pt1)

% Default parameters ----------------

if nargin < 3

tolerance = 0.1; %tolerance for the outliers in terms of x-intercept interval

end

if nargin < 4

xintercep_resolution = 1000; %resolution of the line searching

end

if nargin < 5

xintercept_start = -1; %starting point of the x-intercept range

end

if nargin < 6

xintercept_end = 1; %ending point of the x-intercept range

end

if nargin < 7

leftright = 'right';

else

leftright = 'left';

end

if nargin < 8 % the point (pt1) that should be included the optimal line

if strcmp(leftright, 'right')

[val, ind] = max(x);

else

[val, ind] = min(x);

end

pt1 = [x(ind), y(ind)];

x(ind) = [];

y(ind) = [];

else

ind_x = find(x==pt1(1));

x(ind_x) = [];

end

tf = logical(1);

while tf

for k = 1 : xintercept_resolution

xb = xintercept_start + k*(xintercept_end -

xintercept_start)/xintercept_resolution;

pt2 = [xb, 0];

eqline = point2line(pt1, pt2);

for i = 1 : length(x)

[sdist(k, i), isright(i)] = dist2point(eqline, x(i), y(i));

end

if strcmp(leftright, 'right')

num_side(k) = sum(isright);

svalue(k) = sum(sdist(k, :).*isright);

else

num_side(k) = sum(~isright);

svalue(k) = sum(sdist(k, :).*~isright);

end

end

[minval, minind] = min(svalue);

num_pts = sum(num_side==1);

if num_pts > xintercept_resolution*tolerance/(xintercept_end -

xintercept_start);

[tmp, del_ind] = min(sdist(minind, :));

x(del_ind) = [];

y(del_ind) = [];

else

tf = logical(0);

end

end

pt2m = [xintercept_start+minind*(xintercept_end -...

xintercept_start)/xintercept_resolution, 0];

slope = (pt2m(2) - pt1(2))/(pt2m(1) - pt1(1));

xIntercept = pt2m(1);

function [d, isright, rd] = dist2point(p, x, y)

A = p(1);

B = p(2);

C = p(3);

rd = (A*x + B*y + C)/sqrt(A*A + B*B);

isright = 1;

if rd <= 0

isright = 0;

end

d = abs(rd);

function pset = point2line(p1, p2)

x1 = p1(1);

y1 = p1(2);

x2 = p2(1);

y2 = p2(2);

m = (y2 - y1)/(x2 - x1);

b = y1 - x1*(y2 - y1)/(x2 - x1);

A = m;

B = -1;

C = b;

pset = [A, B, C];

**Supporting Information Section V**: Tables S1 through S3

**Table S1: Envelope parameters**

| Stimuli | Condition | K+H+  Slope | K-H-  Slope | Ratio of  Slopes | K+H+  Intercept | K-H-  Intercept | Intercept  Offset |
| --- | --- | --- | --- | --- | --- | --- | --- |
| Beauty  Keypress ^A^ | Beautiful Female | 3.45 | 2.95 | 1.17 | 0.80 | 0 | 0.80 |
|  | Average Female | 3.13 | 2.57 | 1.22 | 0.63 | -0.23 | 0.86 |
|  | Beautiful Male | 3.81 | 2.75 | 1.39 | 0.81 | -0.1 | 0.91 |
|  | Average Male | 3.29 | 2.50 | 1.32 | 0.66 | -0.26 | 0.92 |
|  | | | | | | | |
| First  IAPS  Set ^B^ | Food | 0 | 5.213 | .00 | -0.998 | 0.592 | -1.59 |
|  | Sports | 0.404 | 4.721 | .09 | -0.998 | 0.606 | -1.60 |
|  | Child | 0 | 2.661 | .00 | -0.998 | 0.278 | -1.28 |
|  | Objects | 0 | 4.743 | .00 | -0.998 | 0.6 | -1.60 |
|  | Nature | 3.433 | 2.406 | 1.43 | 1 | 0 | 1.00 |
|  | Violence | 0 | 1.286 | .00 | -0.998 | -0.998 | .00 |
|  | Disaster | 2.542 | 1.659 | 1.53 | 1 | -0.304 | 1.30 |
|  | Sex | 0 | 2.863 | .00 | -0.998 | 0.304 | -1.30 |
|  | Drugs | 0 | 2.667 | .00 | -0.998 | 0.292 | -1.29 |
| Second  IAPS  Set ^C^ | Food | 23.127 | 4.001 | 5.78 | 1 | 0.54 | .46 |
|  | Sports | 0 | 3.434 | .00 | -0.998 | 0.294 | -1.29 |
|  | Child | 1.164 | 4.226 | .28 | -0.998 | 0.4 | -1.40 |
|  | Objects | 0.433 | 3.886 | .11 | -0.998 | 0.51 | -1.51 |
|  | Nature | 9.152 | 6.64 | 1.38 | 0.956 | 0.742 | .21 |
|  | Violence | 0.786 | 2.08 | .38 | 1 | -0.044 | 1.04 |
|  | Disaster | 0.993 | 1.419 | .70 | 1 | -0.726 | 1.73 |
|  | Sex | 0 | 3.114 | .00 | -0.998 | 0.416 | -1.41 |
|  | Drugs | 0 | 1.451 | .00 | -0.998 | -0.692 | -.31 |

Legend: Power law scaling of the value function was performed with the following three groups: 77^A^ healthy controls for Beauty pictures (see Table 2 legend for numbers of subjects fit), 26^B^ healthy controls for the first set of IAPS pictures (out of 33) and 23^C^ healthy controls for the second set of IAPS pictures (out of 31). These numbers reflected the number of individuals who met criteria for automated fitting using Matlab procedures (see procedures outlined in Supporting Information Section IV). The slopes are expressed in units of keypress number over bits, and the ratio of slopes is dimensionless; the ratio is quantified to assess if it has a narrow range across experimental conditions, which may represent a constant in the relationship of approach and avoidance. The intercept is expressed in units of keypress number (i.e., the x axis value).

**Table S2a:** Beauty Keypress Log_10_KH Linear Regression Parameters

| Variables |  | |
| --- | --- | --- |
|  | Parameters | Mean±SD |
|  | r | 0.93±.24 |
|  | r^2^ | 0.92±.18 |
|  | Adjust r^2^ | 0.87±.31 |
|  | *p* value of r | 0.06±.15 |
|  | *Conjunction p* value of r | 1.39e-162 |
|  | Slope | 2.60±.86 |
|  | X Intercept | 0.40±7.20 |
|  | r | 0.92±.15 |
|  | r^2^ | 0.86±.21 |
|  | Adjust r^2^ | 0.78±.32 |
|  | *p* value of r | 0.11±.16 |
|  | *Conjunction p* value of r | 7.17e-068 |
|  | Slope | 2.35±.94 |
|  | X Intercept | -0.56±1.77 |
|  | Ratio of Slopes | 0.90±.50 |
|  | Intercept Offset | -.04±1.48 |

Legend: fitting was performed on an individual basis with the 77 healthy volunteers keypressing for the Beauty pictures. From this total cohort, 73 subjects and 46 subjects for negative and positive keypress data, respectively, met criteria for automated linear fitting using Matlab procedures; each subject required more than two of four conditions have non-zero results. The linear correlation between the observed and model-predicted values of the Beauty keypress dependent variable is represented by r. The coefficient of determination, r square, is the squared value of the multiple correlation coefficient. Adjusted r square is a "corrected" r square statistic that penalizes models with large numbers of parameters. The slopes are expressed in units of bits over number of keypress and the ratio of slopes is dimensionless; the ratio is quantified to assess if it has a narrow range across experimental conditions, which may represent a constant in the relationship of approach and avoidance. The intercept is expressed in units of keypress number.

**Table S2b:** IAPS and Food Log_10_KH Linear Regression Parameters

| Variables | Parameters | IAPS | | |  | FOOD |
| --- | --- | --- | --- | --- | --- | --- |
|  |  | Stimuli | N | Mean±SD. |  | Mean±SD. |
|  | r | First | 26 | 0.96±.05 |  | 0.98±.01 |
|  |  | Second | 23 | 0.94±.07 |  |  |
|  | r^2^ | First | 26 | 0.93±.09 |  | 0.96±.03 |
|  |  | Second | 23 | 0.89±.13 |  |  |
|  | Adjust r^2^ | First | 26 | 0.91±.11 |  | 0.95±.03 |
|  |  | Second | 23 | 0.87±.15 |  |  |
|  | *p* value of r | First | 26 | 3x10^-3^ ± .01 |  | 1×10^-4^±.0002 |
|  |  | Second | 23 | 0.007±.01 |  |  |
|  | *Conjunction p* value of r | First | 26 | 5.03e-113 |  | 6.23e-028 |
|  |  | Second | 23 | 6.40e-081 |  |  |
|  | Slope | First | 26 | 2.52±.51 |  | 2.53±.28 |
|  |  | Second | 23 | 2.65±.64 |  |  |
|  | X Intercept | First | 26 | -0.02±.31 |  | -0.93±.23 |
|  |  | Second | 23 | 0.01±.34 |  |  |
|  | r | First | 25 | 0.94±.08 |  | 0.95±.03 |
|  |  | Second | 21 | 0.94±.09 |  |  |
|  | r^2^ | First | 25 | 0.90±.13 |  | 0.91±.06 |
|  |  | Second | 21 | 0.88±.15 |  |  |
|  | Adjust r^2^ | First | 25 | 0.87±.18 |  | 0.89±.07 |
|  |  | Second | 21 | 0.86±.18 |  |  |
|  | *p* value of r | First | 25 | 0.02±.08 |  | 6×10^-4^±.0009 |
|  |  | Second | 21 | 0.007±.01 |  |  |
|  | *Conjunction p* value of r | First | 25 | < 4.32e-102 |  | 8.64e-024 |
|  |  | Second | 21 | 2.82e-078 |  |  |
|  | Slope | First | 25 | 2.34±1.09 |  | 2.31±.46 |
|  |  | Second | 21 | 2.16±.80 |  |  |
|  | X Intercept | First | 25 | 0.26±.59 |  | -0.80±.39 |
|  |  | Second | 21 | 0.2±.65 |  |  |
|  | Ratio of Slopes | First | 25 | 0.99±.67 |  | 0.91±.11 |
|  |  | Second | 21 | 1.00±51 |  |  |
|  | Intercept Offset | First | 25 | 0.28±.58 |  | 0.13±.30 |
|  |  | Second | 21 | 0.39±43 |  |  |

Legend: fitting was successfully performed on an individual basis with three sets of subjects: 23-26 healthy volunteers for IAPS first experiment, 21-25 healthy volunteers for IAPS second experiment, and 6 healthy volunteers from the alliesthesia (i.e., FOOD) experiment. These subjects met criteria for automated linear fitting using Matlab procedures; each subject required at least three conditions have non-zero results. The linear correlation between the observed and model-predicted values of the Keypress dependent variable is represented by r. The coefficient of determination, r square, is the squared value of the multiple correlation coefficient. Adjusted r square is a "corrected" r square statistic that penalizes models with large numbers of parameters. The slopes are expressed in units of bits over number of keypress and the ratio of slopes is dimensionless; the ratio is quantified to assess if it has a narrow range across experimental conditions, which may represent a constant in the relationship of approach and avoidance. The intercept is expressed in units of keypress number.

**Table S3a:** Beauty Keypress Quadratic Regression Parameters

| Variables | Parameters | Statistic |
| --- | --- | --- |
|  | r | 0.95±.09 |
|  | r^2^ | 0.90±.16 |
|  | Adjust r^2^ | 0.71±.48 |
|  | *p* value of r | 0.32±.26 |
|  | *Conjunction p* value of r | <1.3491e-092 |
|  | r | 0.97±.07 |
|  | r^2^ | 0.95±.11 |
|  | Adjust r^2^ | 0.86±.33 |
|  | *p* value of r | 0.15±.20 |
|  | *Conjunction p* value of r | <1.4734e-307 |

Legend: quadratic fitting was performed with 77 healthy volunteers who participated in the Beauty experiment. From this total cohort, 65 subjects for fitting and 46 subjects for fitting, respectively, met criteria for automated quadratic fitting using Matlab procedures; each subject required more than two of four conditions have non-zero results. The correlation between the Beauty Keypress observed and model-predicted values of the Beauty Keypress dependent variable is represented by r. The coefficient of determination, r square, is the squared value of the multiple correlation coefficient. Adjusted r square is a "corrected" r square statistic that penalizes models with large numbers of parameters.

**Table S3b:** IAPS and Food Quadratic Regression Parameters

| Variables | Parameters | IAPS | | |  | FOOD |
| --- | --- | --- | --- | --- | --- | --- |
|  |  | Group | N | Mean±SD. |  | Mean±SD. |
|  | r | First | 26 | 0.90±.12 |  | 0.91±.06 |
|  |  | Second | 23 | 0.91±.11 |  |  |
|  | r^2^ | First | 26 | 0.82±.18 |  | 0.82±.11 |
|  |  | Second | 23 | 0.85±.17 |  |  |
|  | Adjust r^2^ | First | 26 | 0.76±.24 |  | 0.75±.15 |
|  |  | Second | 23 | 0.79±.23 |  |  |
|  | *p* value of r | First | 26 | 0.25±.23 |  | 0.16±.19 |
|  |  | Second | 23 | 0.23±.25 |  |  |
|  | *Conjunction p* value of r | First | 26 | 2.87e-031 |  | 5.72e-008 |
|  |  | Second | 23 | 2.28e-027 |  |  |
|  | r | First | 25 | 0.95±.05 |  | 0.91±.08 |
|  |  | Second | 21 | 0.92±.10 |  |  |
|  | r^2^ | First | 25 | 0.90±.09 |  | 0.83±.13 |
|  |  | Second | 21 | 0.86±.17 |  |  |
|  | Adjust r^2^ | First | 25 | 0.87±.12 |  | 0.76±19 |
|  |  | Second | 21 | 0.81±.22 |  |  |
|  | *p* value of r | First | 25 | 0.17±.26 |  | 0.06±.08 |
|  |  | Second | 21 | 0.12±.22 |  |  |
|  | *Conjunction p* value of r | First | 25 | < 2.06e-062 |  | 3.15e-014 |
|  |  | Second | 21 | 8.72e-035 |  |  |

Legend: fitting was successfully performed on an individual basis with three sets of subjects: 23-26 healthy volunteers for IAPS first experiment, 21-25 healthy volunteers for IAPS second experiment, and 6 healthy volunteers from the alliesthesia (i.e., FOOD) experiment. These subjects met criteria for automated quadratic fitting using Matlab procedures; each subject required at least three conditions have non-zero results (i.e., 3 of 9 conditions for the IAPS, and 3 of 8 conditions for the alliesthesia experiment). The multiple correlation coefficient, r, is the correlation between the observed and model-predicted values of the dependent variable. The coefficient of determination, r square, is the squared value of the multiple correlation coefficient. Adjusted r square is a "corrected" r square statistic that penalizes models with large numbers of parameters.

**Supporting Information Section VI:** Shannon Entropy Computation

In this study, each subject would produce a response profile as shown in Figure 1b. These response profiles were interpreted to be relative frequency (probability) distributions in a Typewriter model. This entailed translating the response profile so that the subject would choose a keypress response to a particular picture with a certain probability on each occasion. In a psychological test, one could present N picture items all at once and let the subject choose a picture at each experimental occasion, repeating this process until statistical power is achieved; alternately, one could make the response profile reflect the relative frequency of each picture in random sequence, just like the Typewriter model works with letter strings in a sequence.

These considerations were encoded within the following definition of an ensemble: an ensemble of a behavioral response profile ‘X’ is a random variable x with a set of possible outcomes, , having probabilities , with $P(x=a_{i})=p_{i}$, and $\sum P(x)=1$. The first order entropy of this ensemble can be computed by the Shannon entropy: , where $H_{+}$is the entropy of increasing keypresses, $H_{-}$ is the entropy of decreasing keypresses, $P_{+,i}$is the relative intensity of the increasing keypresses for the item (economic commodity) *i*, $P_{-,i}$ is the relative intensity of the decreasing keypresses for the item (economic commodity) *i*, and N is the number of the alternatives. The first order entropy applies to this data source since it is structured by the probability distribution of independent or differentiable events. The value of an element of our data set (e.g., the number of keypresses for the 7th beautiful female face or other picture shown) is proportional to the probability of events associated with the element (how likely the subject would respond to the stimuli) which is a measure of the predictability of the behavior associated with the stimulus conditions as well as the irregularity of the data set. For cases where subjects made no keypress responses, but accepted the default condition for N of N items in the itemset for a condition, we defined H = 0. In the theoretical context that the number of items in a category of economic commodity, $x_{p}$, was decreased to 1, $H_{\pm}$ would also approach 0, meaning there was no ambiguity in choice. This information theoretic approach [64,66,67,137-143] is grounded in the classical theory of entropy, but does not necessitate the temporal evolution of an ensemble.

To illustrate how these computations were performed, consider the increasing key response variable for a set of beautiful female faces (BF) from one experimental subject, seen in the key response profiles for the pink blocks below:


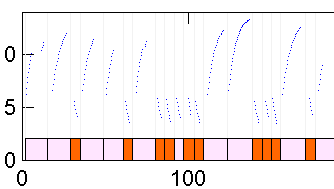

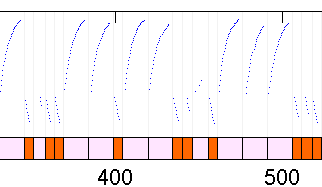


Step 1: The number of increasing keypresses (time-wise) = {40, 54, 47, 31, 43, 0, 61, 96, 69, 20, 86, 0, 88, 79, 87, 73, 9, 91, 69, 78}

Step 2: The number of increasing keypresses (face identity-wise) = {96, 43, 20, 54, 0, 47, 69, 31, 61, 40, 91, 73, 9, 86, 79, 88, 0, 69, 78, 87}

Step 3: The ratio of the number of the increasing keypresses (ratio, face identity-wise) = {0.0856, 0.0384, 0.0178, 0.0482, 0, 0.0419, 0.0616, 0.0277, 0.0544, 0.0357, 0.0812, 0.0651, 0.0080, 0.0767, 0.0705, 0.0785, 0, 0.0616, 0.0696, 0.0776}

H(**BF**) = = 4.0312 bit for the increasing keypresses (i.e., you need ~ 4 bits of information for each BF face to describe the subject’s intra-condition behavior.
