# Supplementary figures and images for: Recurrent, Robust and Scalable Patterns Underlie Human Approach and Avoidance
Source: PLoS One. 2010 May 26;5(5):e10613. doi: 10.1371/journal.pone.0010613 (PMC2879576; doi:10.1371/journal.pone.0010613)

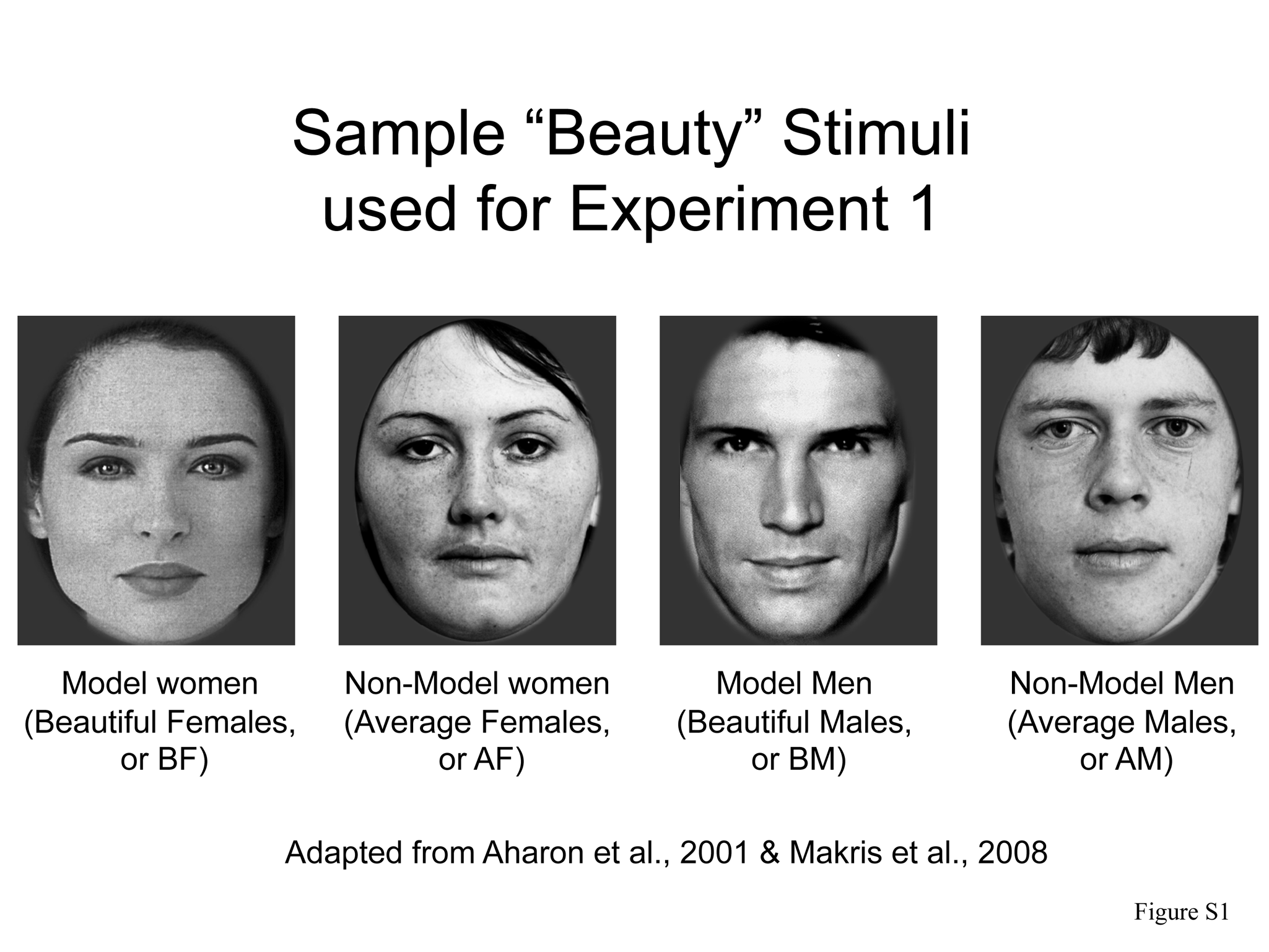

Supplement: Figure S1 — Examples of Beauty Stimuli. A sample of the four picture types used for the beauty stimuli (from left to right): beautiful female, average female, beautiful male and average male. Each of these experimental conditions or categories of picture consisted of either 20 male or 20 female faces. Since initial development (see acknowledgments and [14], [29]), these stimuli have been used in a number of studies [16], [17], [19]. (1.06 MB TIF) [file pone.0010613.s002.tif]

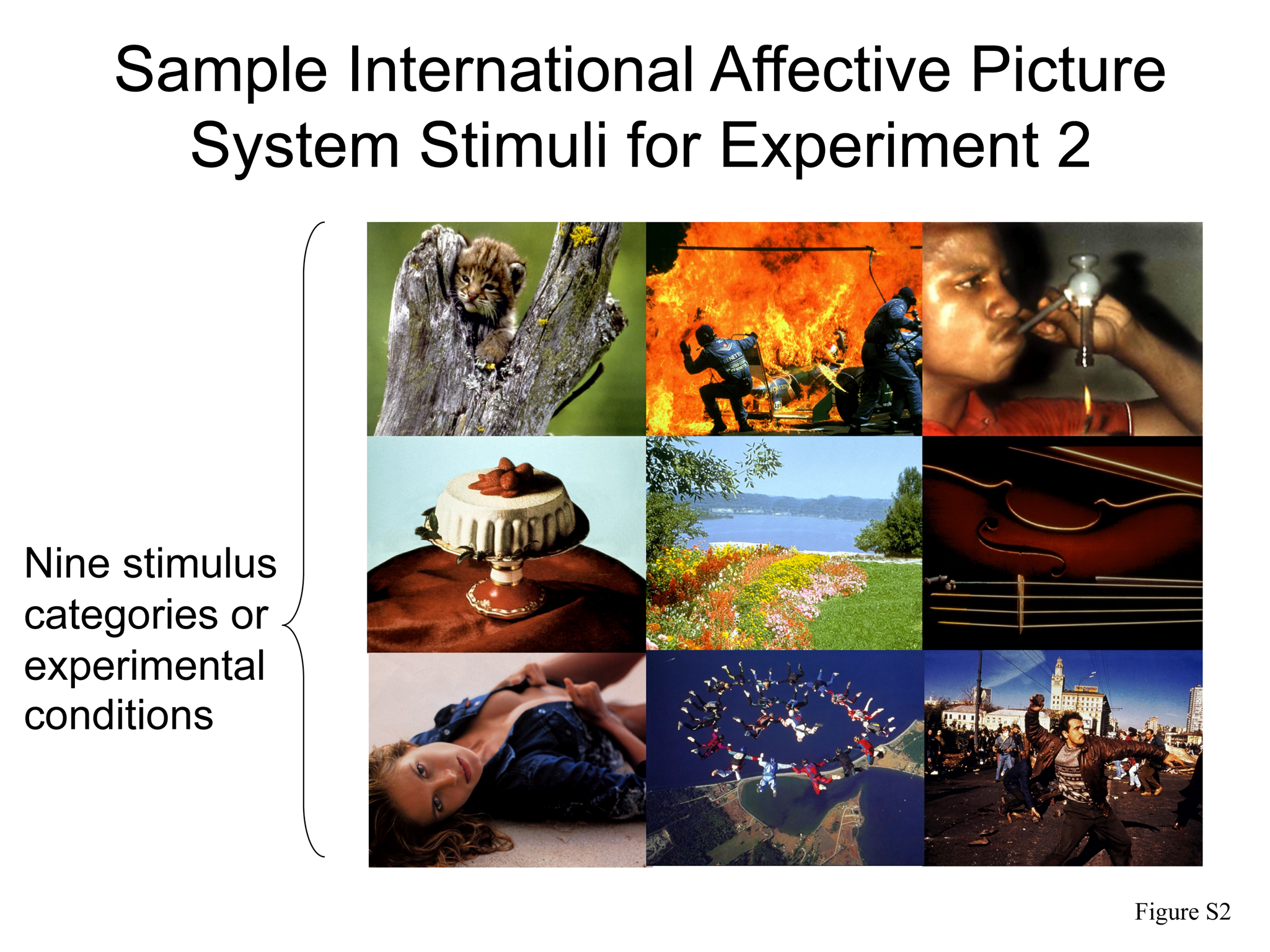

Supplement: Figure S2 — Representative pictures from the International Affective Picture System (IAPS) [61], [62]. Images used came from nine distinct categories of picture content: objects, nudes/sex, sports, disasters, food, kids/pets, nature, violence/war, and drug paraphernalia. Each category contained 9 pictures. Please see Methods, Picture Stimuli for Keypress Experiments, for further information and commentary. (3.01 MB TIF) [file pone.0010613.s003.tif]

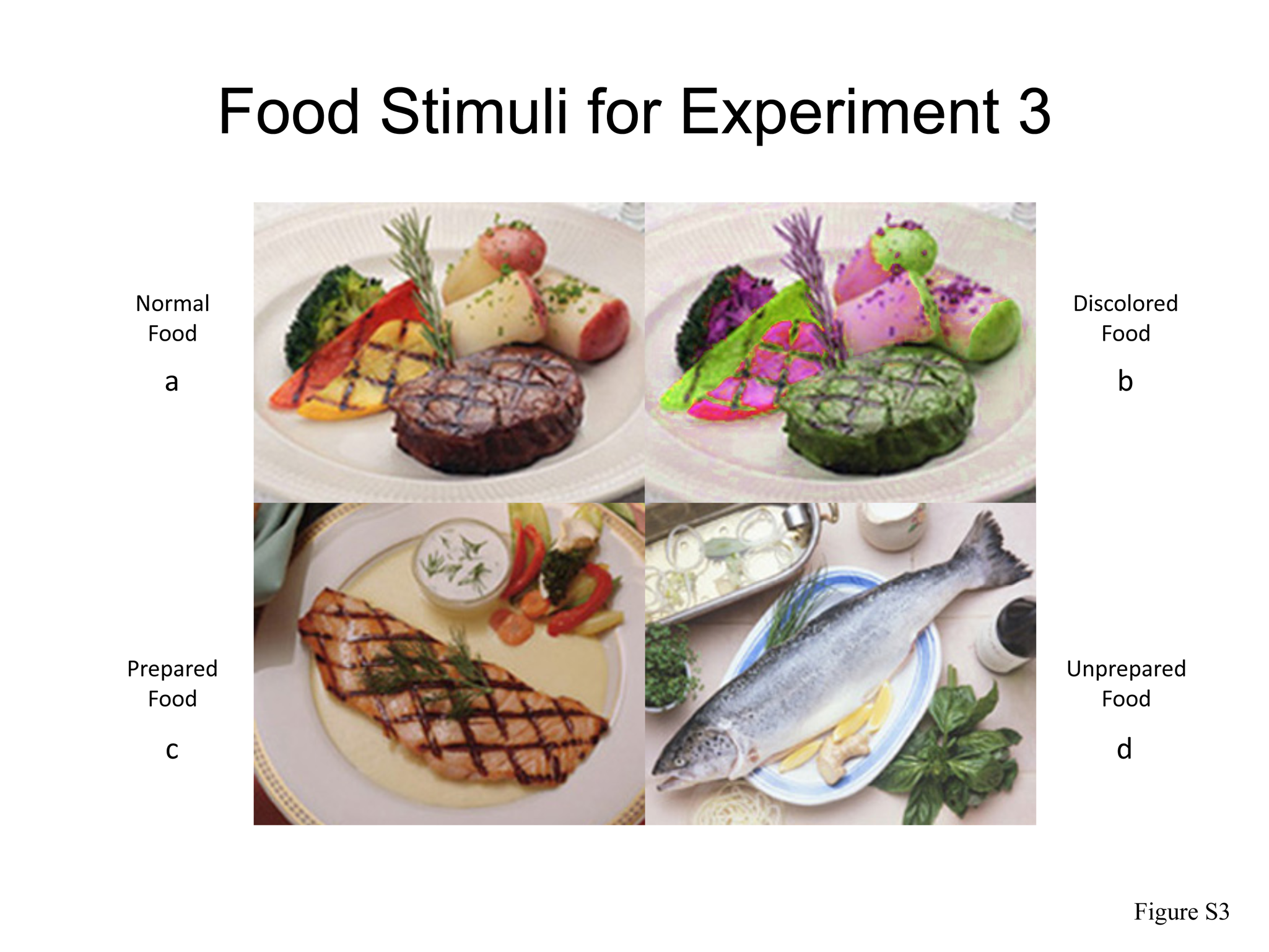

Supplement: Figure S3 — Examples of Food Stimuli. One example of items from each category of food stimuli: (a) Normally colored food item; (b) Discolored food item; (c) Prepared food item; (d) Unprepared food item. (2.07 MB TIF) [file pone.0010613.s004.tif]

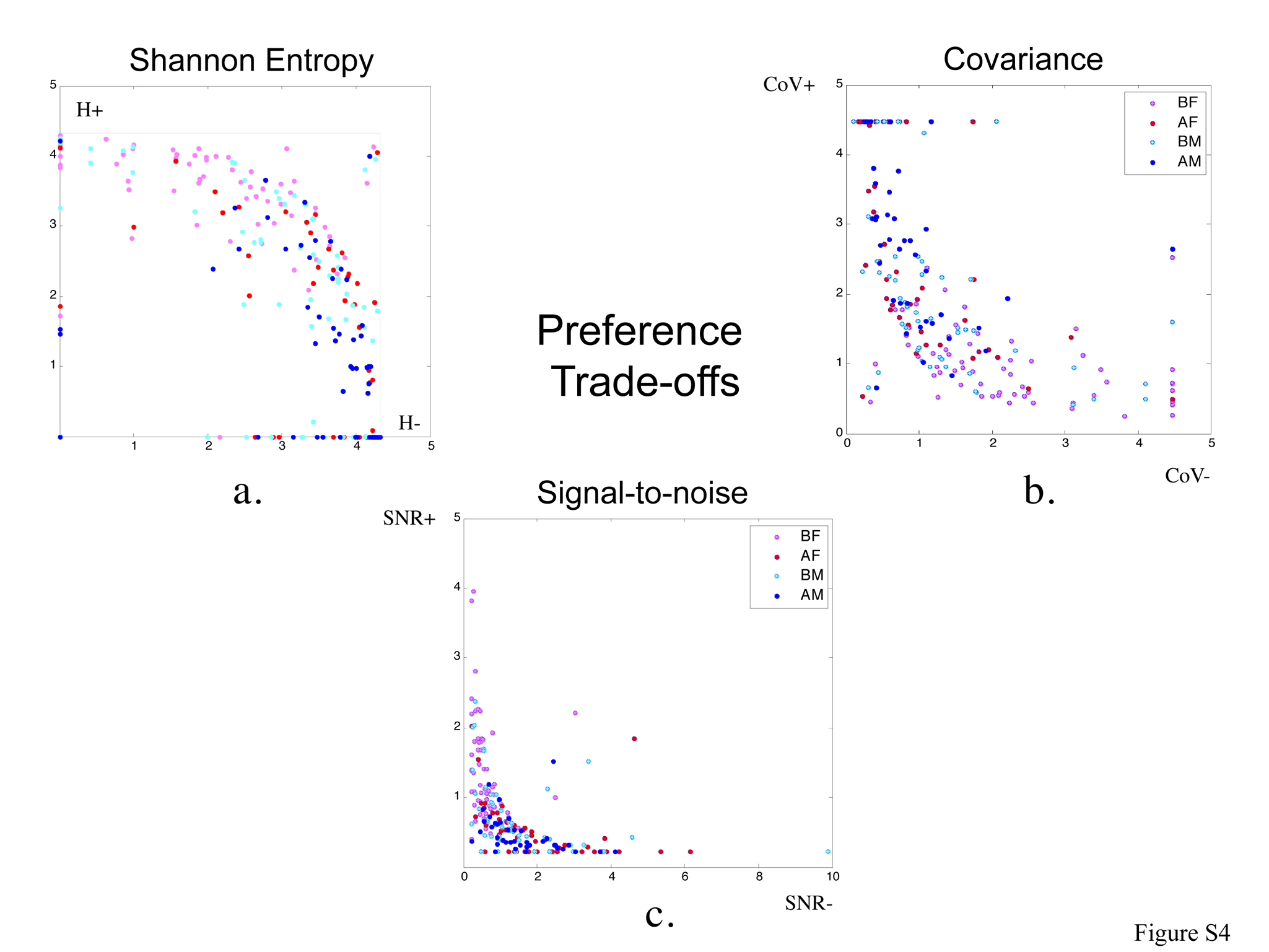

Supplement: Figure S4 — Examples of Trade-off plots Using Pattern-variables. Three functionally similar types of preference trade-off graphs are displayed for H, SNR, and CoV estimates. In (a), a graph is displayed of the Shannon entropy for increasing keypress responses (y axis) versus the entropy for decreasing keypress responses (x axis) for responses to BF, AF, AM, AM faces in 77 healthy control subjects. For the same set of experimental subjects, we show in (b) a graph for the manifold, and in (c) a graph for , which represents a boundary envelope. (0.25 MB TIF) [file pone.0010613.s005.tif]

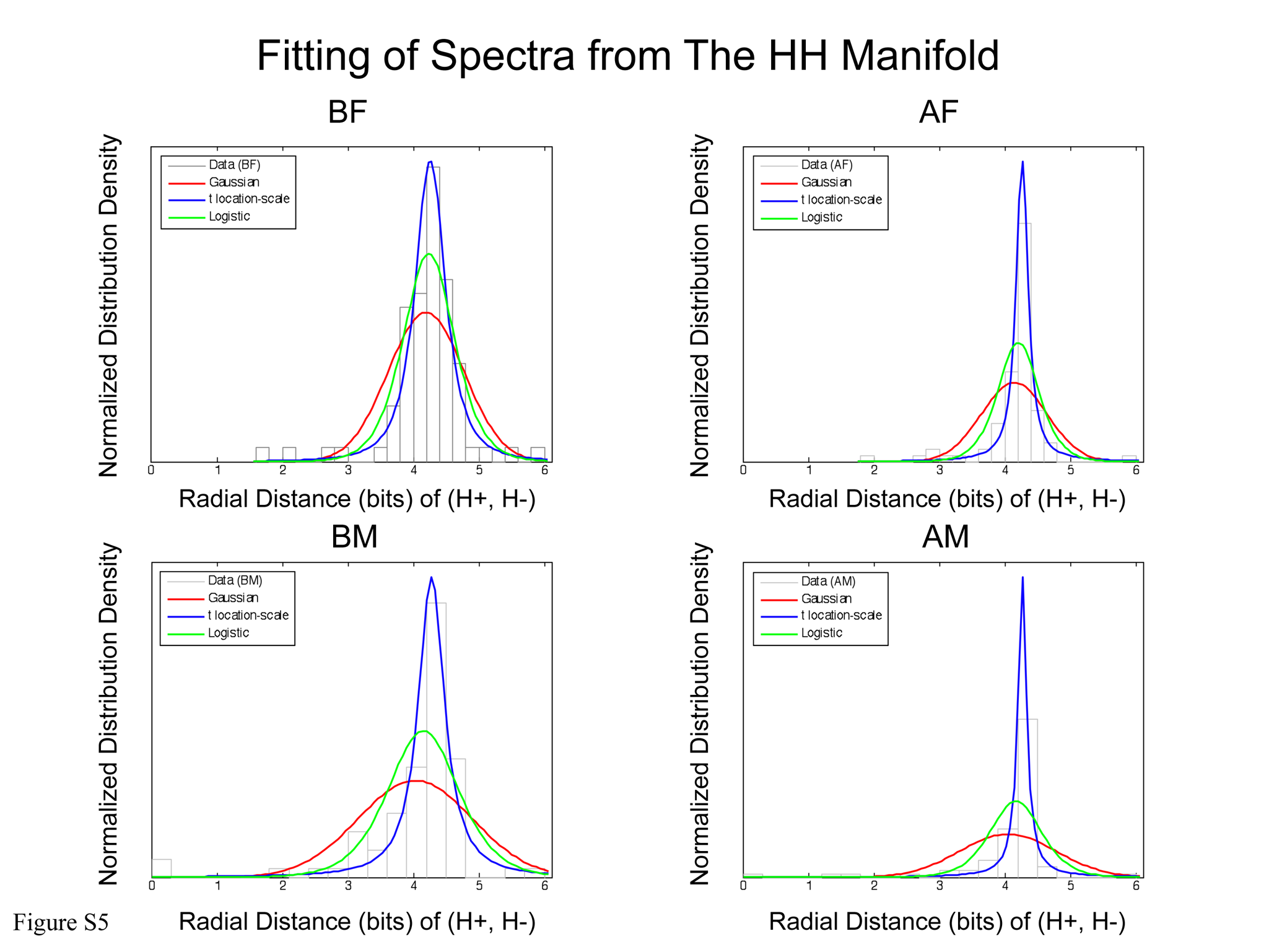

Supplement: Figure S5 — Radial Distributions from Trade-off Plots. Each graph represents the data from 77 healthy controls, for one experimental condition (i.e., BF, AF, BM, AM faces), with three types of fitting of the radial distribution from the trade-off plot of that experimental condition. Radial sampling of the preference trade-off graphs for these four experimental conditions were tabulated using bins of 0.2 bits (in gray-tone lines off the x-axis). Bin height reflected the normalized number of data points across 77 subjects. Fitting through three methods (see Supporting Information File S1 Section I) was performed, so that each of the resulting curves contains the same area or number of samples. Qualitatively, the best fit is observed with the t location-scale distribution. (0.46 MB TIF) [file pone.0010613.s006.tif]

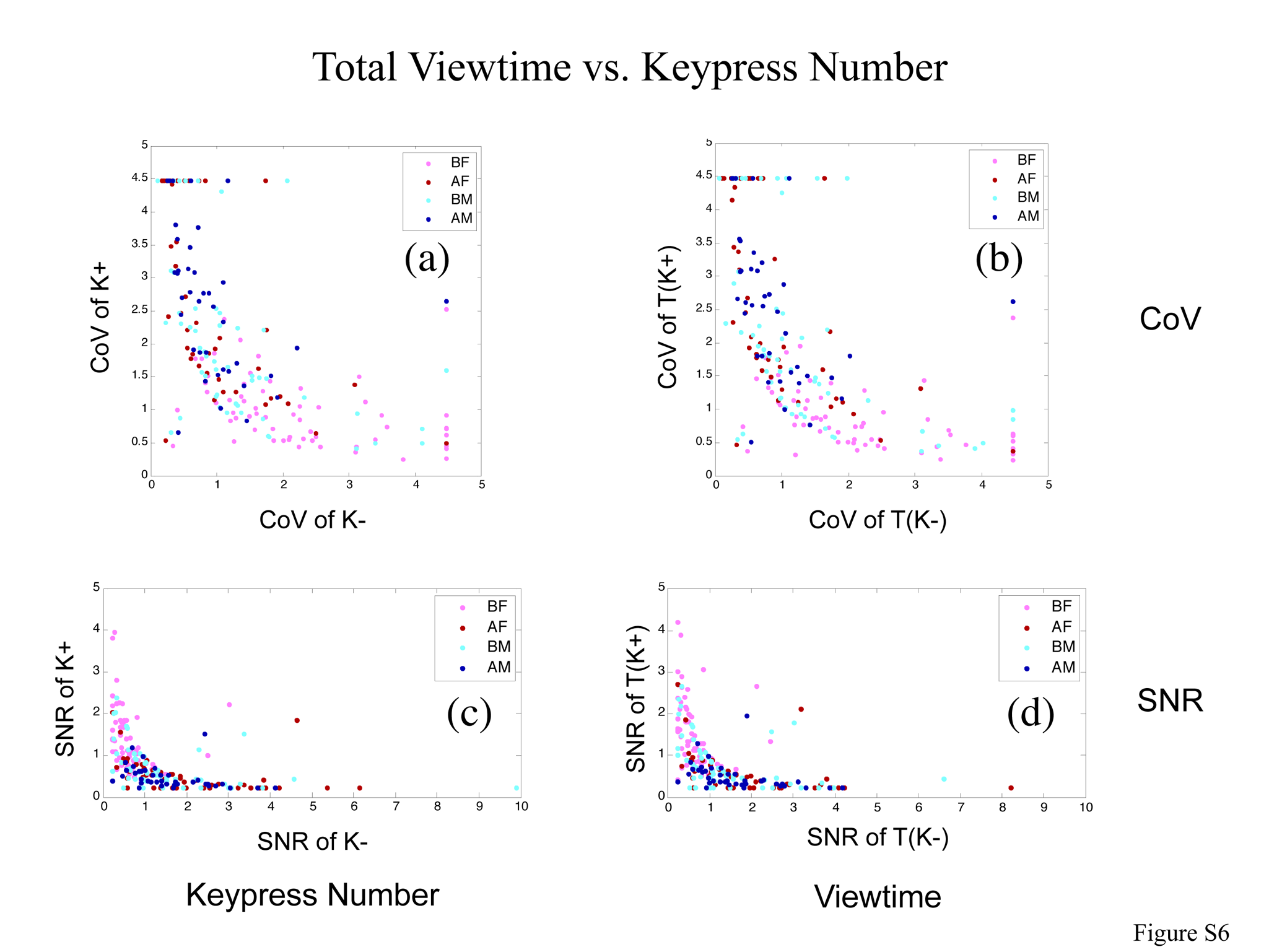

Supplement: Figure S6 — Trade-off Plots for Total Viewtime Versus Keypress Number. The resistive function used to translate keypress effort into viewing time theoretically might influence the form of the preference trade-off, the value function or the saturation function. To rule this out, we analyzed total viewtime data (symbolized by T(K+) or T(K−) for viewtimes resulting from pressing the positive keys or the negative keys respectively), to determine if the same set of patterns was observed with group data, or whether there were discrete functions with individual data. The resulting graphs of the preference trade-off, value function, and saturation function exhibited the same patterns whether or not using keypress number or viewtime data. In this figure, the manifold is shown for keypress data (a) and for viewtime data (b). To further support the observation shown in Figure S4, the same comparison of keypress versus total viewtime data (again using the symbolization of T(K+) or T(K−) for viewtimes resulting from pressing the positive keys or the negative keys respectively) is shown using SNR estimates. The plot is shown for keypress number (c) and total veiwtime (d). This observation further supports the potential for these analyses to be used for other frequency data besides that acquired using keypress procedures. (0.32 MB TIF) [file pone.0010613.s007.tif]

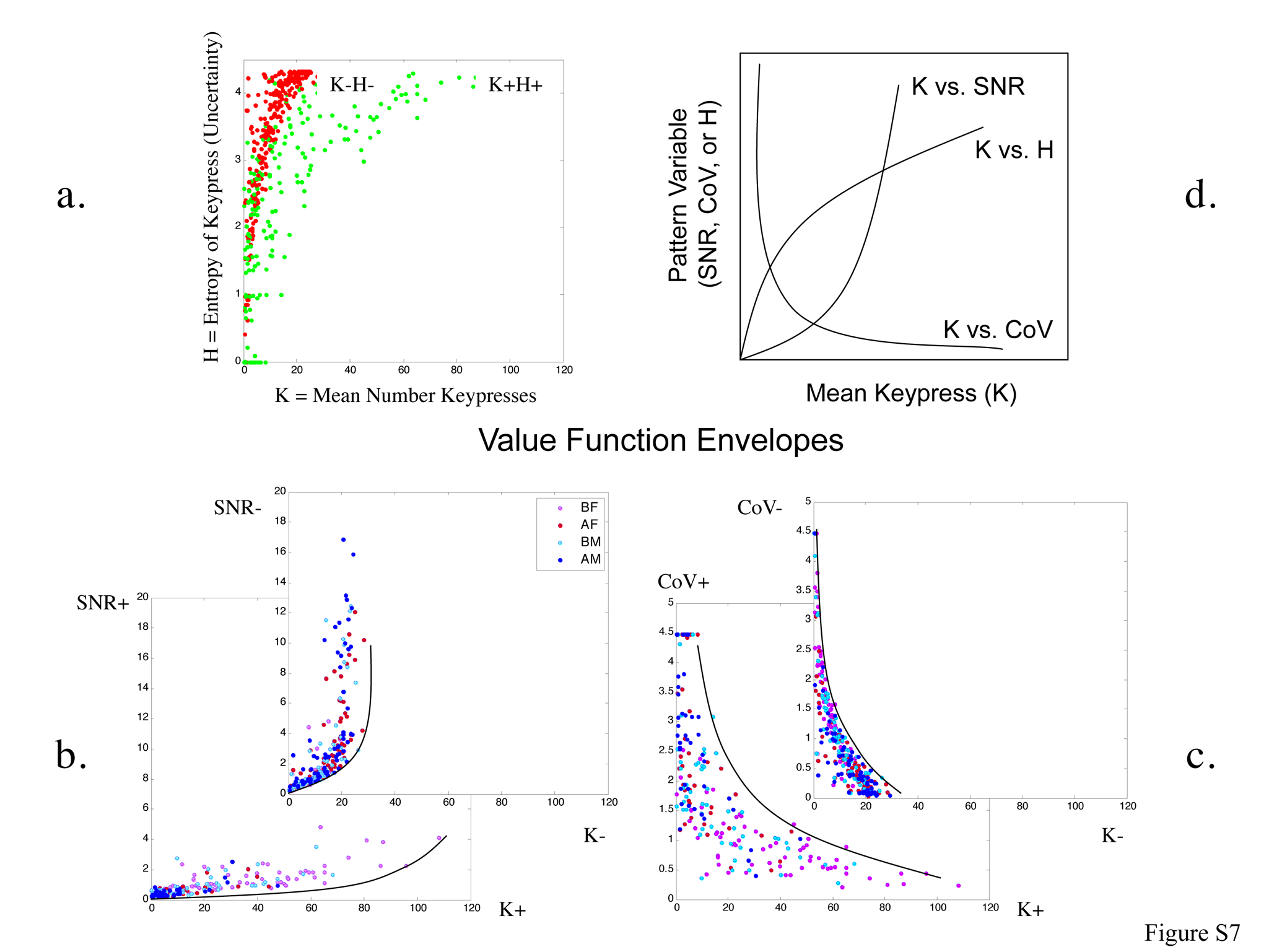

Supplement: Figure S7 — Examples of Value Function Graphs Using Pattern-variables. Three types of value function graphs are displayed for H, SNR, and CoV estimates. In (a), graphs of are shown in red for the negative (avoidance) keypress and green for the positive (approach) keypress for 77 healthy controls, with no color-coding between BF, AF, BM, AM stimulus conditions. The approach and avoidance keypress data are displayed on the same axes to illustrate the difference in curvature between approach and avoidance responses, which approximates the observation of “loss aversion” described in prospect theory. Similar differences between approach and avoidance slopes are also observed for value functions using SNR and CoV estimates. The boundary envelopes for graphs (b), and for graphs (c) are also shown for BF, AF, BM, AM faces in 77 healthy control subjects. In (d), a cartoon of the differences in boundary envelopes observed across value functions with H, SNR, and CoV estimates is illustrated. Note that a similar graphical structure to that observed with the plot is also observed with a very different psychological phenomenon, namely the Weber-Fechner-Stevens Law in sensory psychophysics [146]–[150], underscoring the pervasiveness of power functions in nature [68]–[72]. (0.37 MB TIF) [file pone.0010613.s008.tif]

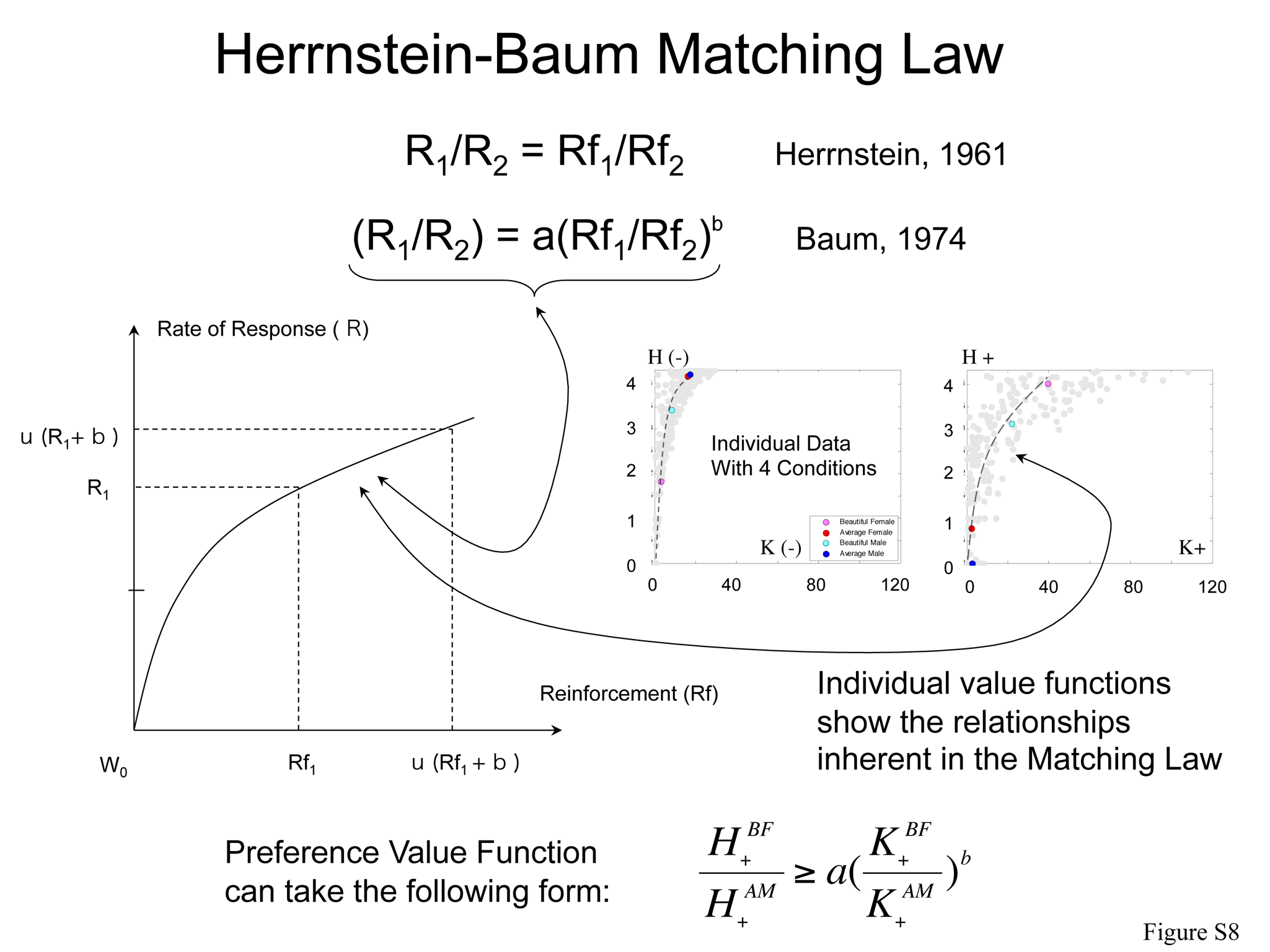

Supplement: Figure S8 — Comparing the Value Function with the Matching Law. The matching law as described by Herrnstein [39] was initially approximated by ratios. Later work by Baum [40] suggested that matching could be better described by a power function, although modern research regarding matching works elegantly with the initial formulation of Herrnstein [39]. These issues are of interest given the observation of power law scaling with the value function, which allows ratios to be represented with the same mathematical structure (equation at bottom) for individual data (two plots on right). (0.35 MB TIF) [file pone.0010613.s009.tif]

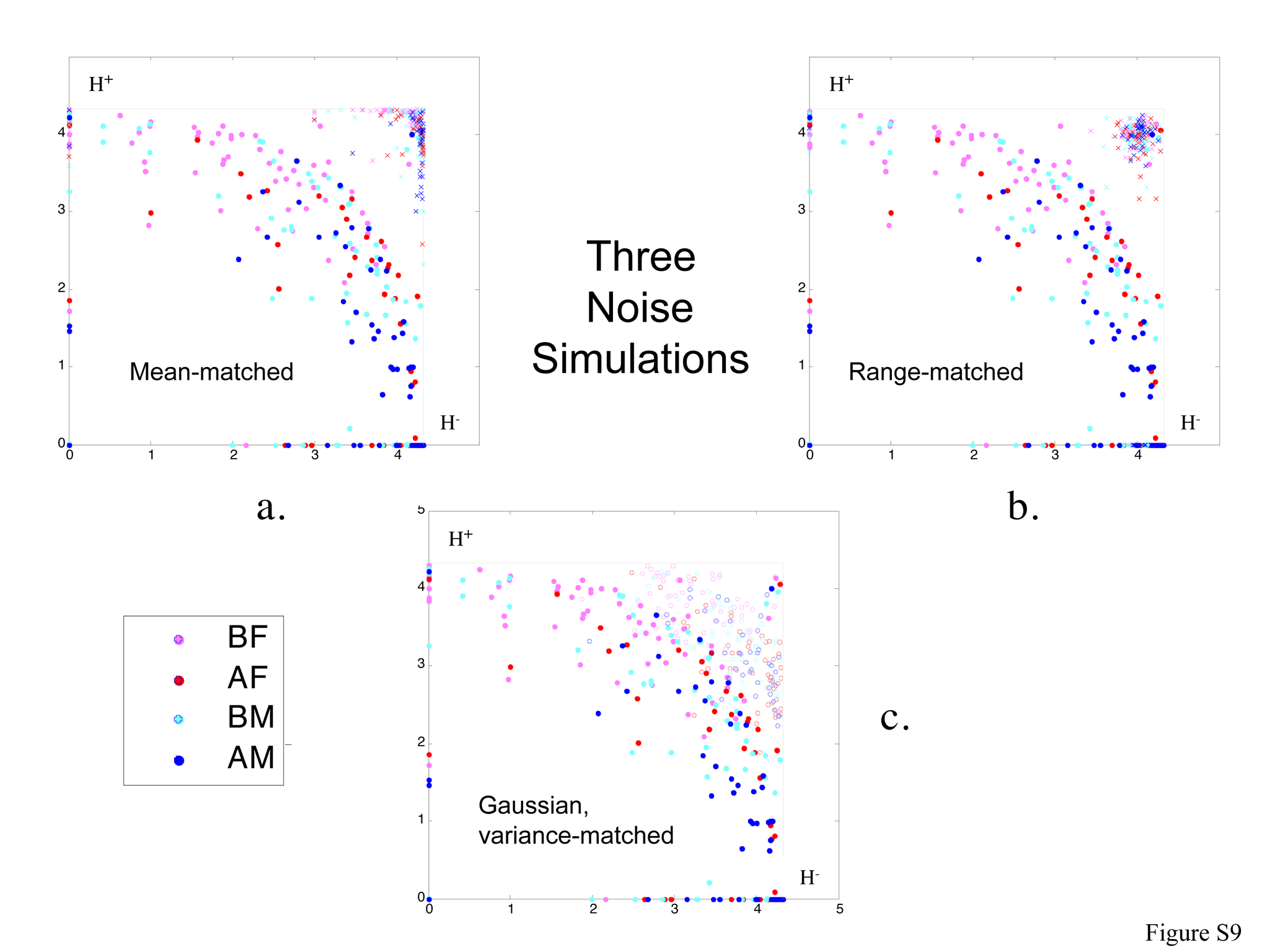

Supplement: Figure S9 — Noise Simulations. Three noise simulations were run for each of the four experimental conditions, and combined in the illustrated graphs (real subject data with filled circles, and hypothetical subject data with x's or open circles). These three simulations include: (a) mean-matched uniform random noise, (b) range-matched uniform random noise, and (c) variance-matched Gaussian noise. Procedures for these simulations are described in the main text methods section. Note that across these three graphs, no simulation duplicates the human experimental data, or a subset thereof. (0.30 MB TIF) [file pone.0010613.s010.tif]

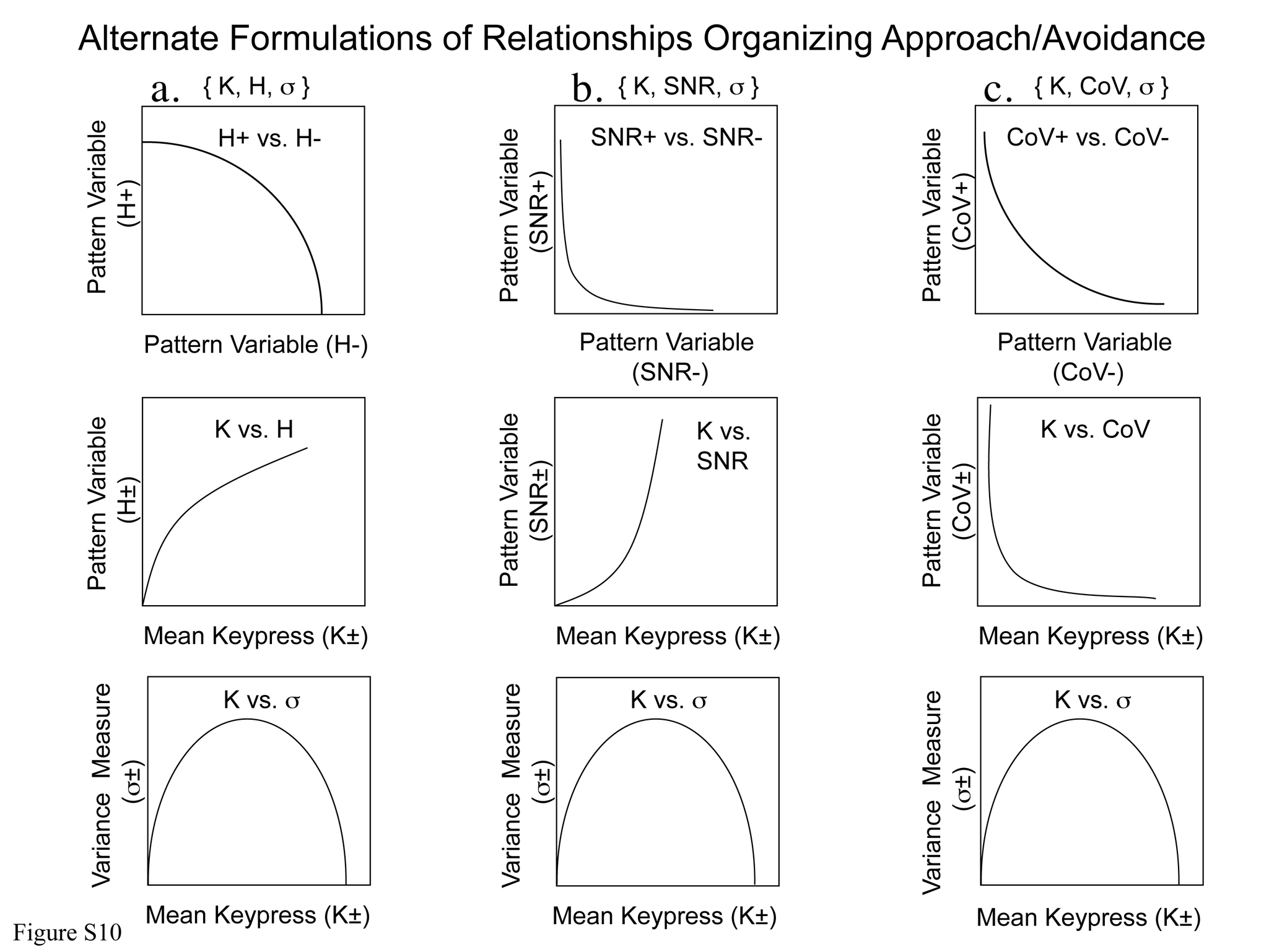

Supplement: Figure S10 — Alternate Sets of Patterns Characterizing Approach and Avoidance. There appear to be at least three alternate formulations of the relationships organizing relative preference in humans. These three formulations are illustrated schematically in three columns of graphs, with a trade-off relationship on top of each, a value function in the middle, and a saturation function on the bottom. With group data, the trade-off relationships represent manifolds for the plot (a) and plot (c), and a boundary envelope for the plot (b). The central tendency of the plot has a similar mathematical form to the graphs of individual data across experimental conditions tested, although there can be significant variability across individuals. For all of the value functions assessed, group data reveals an envelope for the , , and plots. In individuals, plots reveal striking functional fits. Lastly, one can associate the plot with each of the graphs produced using the three pattern variables. (0.39 MB TIF) [file pone.0010613.s011.tif]

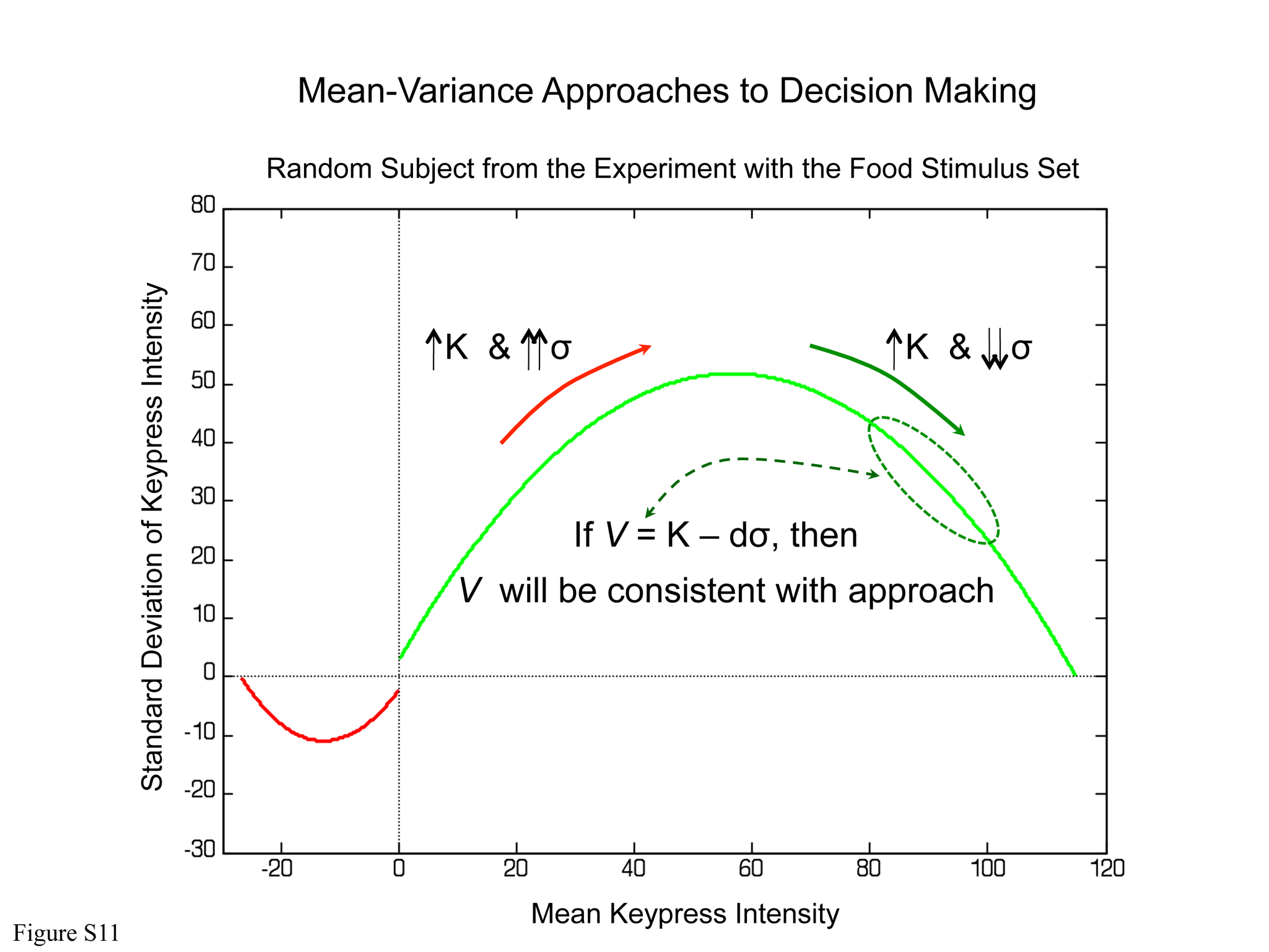

Supplement: Figure S11 — Plot and Mean-Variance Model of Choice. The plot may have relevance for mean-variance approaches to decision making under risk. As described by D'Acremont and Bossaerts [78], the mean-variance approach describes risk by the outcome variance (i.e., standard deviation, ), and computes a valuation V by the difference between the mean transaction outcome and variance estimate: V , where d is the penalty imposed for risk. As d increases, the individual shows increasing risk aversion. In the quadratic fitting of , the computation of V is not likely to show that the individual prefers mappings on the plot until after has reached a maximum and is decreasing (while K continues to increase). This might not be necessary if d is quite low, in which case one could imagine preferred choices being represented on the plot by mappings with low , and either high or low K. Given the plot involves both approach (positive) and avoidance (negative) components, one might also imagine adapting the mean-variance framework to include both components in the valuation computation (e.g., so that , , , and are all incorporated in the computation). (0.33 MB TIF) [file pone.0010613.s012.tif]
